# Effect of temperature on the structure, catalyst and magnetic properties of un-doped zinc oxide nanoparticles: experimental and DFT calculation†

**DOI:** 10.1039/d4ra04252b

**Published:** 2024-09-30

**Authors:** Masoomeh Sharbatdaran, Mehdi Janbazi

**Affiliations:** a Physics and Accelerators School, Nuclear Sciences and Technology Research Institute Karaj Iran msharbatdaran@aeoi.org.ir +982634256370

## Abstract

Zinc oxide nanoparticles were synthesized using sol–gel and hydrothermal techniques and characterized at different calcination temperatures (400, 500, and 600 °C). The study included an analysis of morphology, crystalline phase, particle size, elemental analysis, specific surface area and chemical state. Various characterization methods were employed, including scanning electron microscopy (SEM), transmission electron microscopy (TEM), X-ray powder diffraction (XRD), surface analysis (BET), nitrogen absorption and desorption (N_2_-desorption), Fourier transform infrared spectroscopy (FTIR), thermal analysis (TGA-DSC), temperature-programmed reduction of hydrogen (H_2_-TPR). Additionally, magnetic properties ZnO nanoparticles were investigated by electron spin resonance (ESR). The investigation revealed changes in reduction behavior, electron spin states, and magnetic properties. The interplay between defects, crystallization, and stability underscores the complexity of ZnO-NPs. These findings contribute to our understanding of nanomaterials and their potential applications in various fields. Density Functional Theory (DFT) calculations with a Hubbard U correction were performed to investigate native defects in ZnO and ZnOCH structures under oxygen-poor (low temperature), oxygen-rich (high temperature) and equilibrium (average temperature) conditions. The formation energies of native defects were calculated, and ESR spectra were simulated to analyze the presence and absence of C=O, C–O, CH, and OH bands, as well as to identify the native defects present during growth. The results of the formation energy calculations and the simulated ESR spectra showed that the growth environment influences the native defects that occur during the ZnO preparation process. Inconsistencies between the calculation of formation energy and the ESR spectra suggested that the C=O, C–O, CH, and OH bands were negligible and could be disregarded in the ZnO nanoparticles. The findings from this study contribute to a deeper understanding of ZnO-NPs, enabling the optimization of their properties for specific applications, such as effective catalysts in chemical reactions.

## Abstract 2

Zinc oxide nanoparticles were synthesized using sol–gel and hydrothermal techniques and characterized at different calcination temperatures (400, 500, and 600 °C).

## Graphical abstract

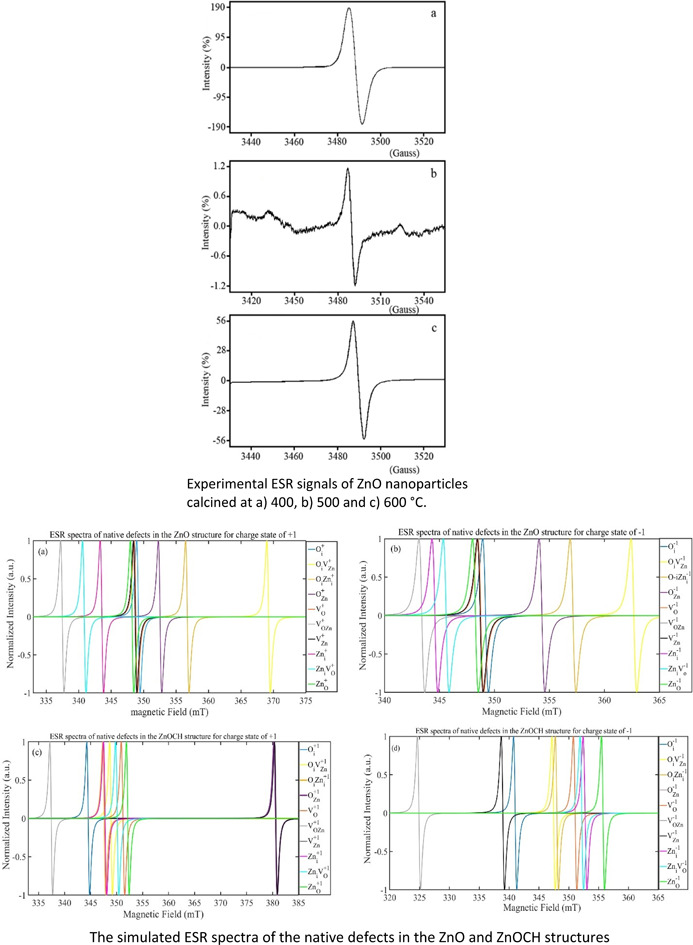

## Introduction

With the emergence of nanotechnology, various inorganic nanostructures like metal nanoparticles and metal oxides have been exploited due to their inherent properties such as optical, magnetic, absorptive and catalytic abilities.^1,2^ Among these materials, zinc oxide nanoparticles have garnered significant attention in both basic and applied research. Zinc oxide belongs to the group of semiconductors and has a wide bandwidth for electron flux, which plays an important role in catalytic activities.^3–5^ On the other hand, zinc oxide is generally known to be a suitable host for a range of modifying elements.^6–9^

Zinc oxide (ZnO) nanoparticles have garnered significant attention in the field of nanotechnology due to their unique properties and versatile applications. With a wide range of potential uses spanning from sunscreen formulation to optoelectronic devices and biomedical applications, ZnO nanoparticles offer a promising avenue for innovation and advancement in various industries.^10–12^ This introduction aims to explore the properties, synthesis methods, and diverse applications of ZnO nanoparticles, shedding light on their significance in the realm of nanomaterials and their potential to drive advancements in technology, healthcare, and environmental remediation.^13,14^

Electron spin resonance, is a powerful technique used for the characterization of materials with unpaired electrons, such as transition metal ions, defects in crystals, and certain organic radicals.^15,16^ When it comes to the characterization of ZnO, ESR can provide valuable insights into the electronic structure and defects present in the material. In the context of ZnO, ESR can be used to study various defects and impurities within the crystal lattice. ZnO is known to exhibit a variety of intrinsic and extrinsic defects, such as oxygen vacancies, zinc interstitials, and various impurities, which can significantly influence its properties and behaviour in different applications.^17–21^

By subjecting ZnO samples to ESR analysis, researchers can observe the behaviour of unpaired electrons in the material when exposed to a magnetic field. This can provide information about the local environment of the defects, their concentration, and their impact on the material's properties, such as its electrical and optical behaviour.^22^ Moreover, EPR can be used to investigate the influence of external factors, such as temperature and irradiation, on the defects within ZnO, offering valuable insights into the stability and dynamics of these defects under different conditions.^23^ In summary, ESR characterization of ZnO can provide crucial information about the nature of defects and impurities within the material, shedding light on its electronic structure and behaviour.^24^ This knowledge is essential for understanding and optimizing the performance of ZnO in various applications, including semiconductor devices,^25^ photocatalysis^26^ and sensors.^27^

ZnO nanoparticles (ZnO-NPs) are indeed suitable for use in optical and magnetic devices due to their unique properties that differ significantly from bulk ZnO.^28^ The specific objectives of studying ZnO-NPs instead of bulk or surface ZnO include the fact that ZnO-NPs exhibit size-dependent properties, such as increased surface area, quantum confinement effects, and altered electronic and optical behaviors. These characteristics make them more suitable for applications in optoelectronic devices, sensors, and catalysts.^29^ The synthesis of ZnO-NPs allows for the introduction and control of defects at the nanoscale, which can significantly influence their magnetic and electronic properties.^30^ Understanding these defects is crucial for optimizing the performance of devices. ZnO-NPs can be tailored for specific applications, such as in photonic devices, where their optical properties can be fine-tuned for better performance.^31^ Their magnetic properties, influenced by defects, can also be harnessed for spintronic applications. The high surface-to-volume ratio of ZnO-NPs enhances their reactivity, making them more effective in catalytic processes compared to bulk ZnO.^32^

Considering that the properties of ZnO nanoparticles prepared by the sol–gel route strongly depend on calcination temperature, we have used different temperature in order to identify and characterize defects in ZnO, such as vacancies, interstitials and concentration of these defects. By studying the ESR spectra of ZnO samples, researchers can gain insights into the nature and, which can impact the material's electronic and optical properties.

In other words, aim of present study was to evaluate the influence of the temperature on the textural, electronic, optical, and magnetic properties of the final sol–gel derived ZnO nano particles. In this context, ZnO nanoparticles was prepared by sol–gel method and after calcination at different temperature, the electronic, optical, and magnetic properties of ZnO structures were investigating in order to provide insights into their fundamental properties and potential applications in various fields.

In this work, a theoretical study using DFT+U was conducted to investigate native defects in ZnO and ZnO–CH, including the presence of C=O, C–O, CH, and OH bands in the ZnO structure under oxygen-poor (low temperature), oxygen-rich (high temperature), and equilibrium (average temperature) conditions. The native defects considered in this supercell included oxygen vacancies (V_O_), interstitial oxygen (O_i_), zinc vacancies (V_Zn_) interstitial zinc (Zn_i_), O at V_Zn_(O_Zn_) and Zn at V_O_(Zn_O_) as well as defect complexes, such as O_i_V_Zn_, Zn_i_V_O_, O_i_Zn_i_, and V_OZn_. The calculated formation energies revealed that the growth environment affected the types of intrinsic defects formed in ZnO and ZnOCH. Ultimately, the ESR spectra were simulated for the native defects to provide further verification of the defect types.

## Experimental

### Preparation of gels

To prepare zinc oxide nanoparticles, a 0.2 M zinc nitrate solution was made and placed in a beaker containing oil. The solution was stirred vigorously at 100 °C for one hour at a speed of 1000 rpm. Meanwhile, 0.435 grams of polyvinyl pyrrolidone (PVP) was dissolved in a 5 ml flask with a mixture of water and ethanol in a 1 : 4 ratio. Additionally, 3 ml of diethylene glycol was prepared. First, PVP was rapidly added to the zinc nitrate mixture, followed by the dropwise addition of diethylene glycol. The resulting solution was stirred at 1000 rpm for two hours, during which it underwent hydrolysis through the slow addition of 50 ml of sodium hydroxide (NaOH) solution. The hydrolysis mixture was stirred vigorously at the same speed, with the rate of NaOH addition and aging time consistently maintained at 20 minutes for all experiments. Mineral-polymeric structures formed after hydrolysis, and a complete stable sol was achieved after allowing two hours following the NaOH addition. The formed sol was then placed in an ultrasonic bath at 80 °C for 30 minutes. Subsequently, it was reheated under the same stirring speed and temperature conditions as before, reaching reflux for 20 hours. Afterward, the sol was transferred to a glass container and subjected to a hydrothermal process at 90 °C for approximately 48 to 72 hours. The solvent was removed by filtration, and the resulting gels were dried at 150 °C overnight. Finally, the dried gels were calcined in air at temperatures of 400, 500, and 600 °C for two hours.

### Computational methods

**Fig. 1:**
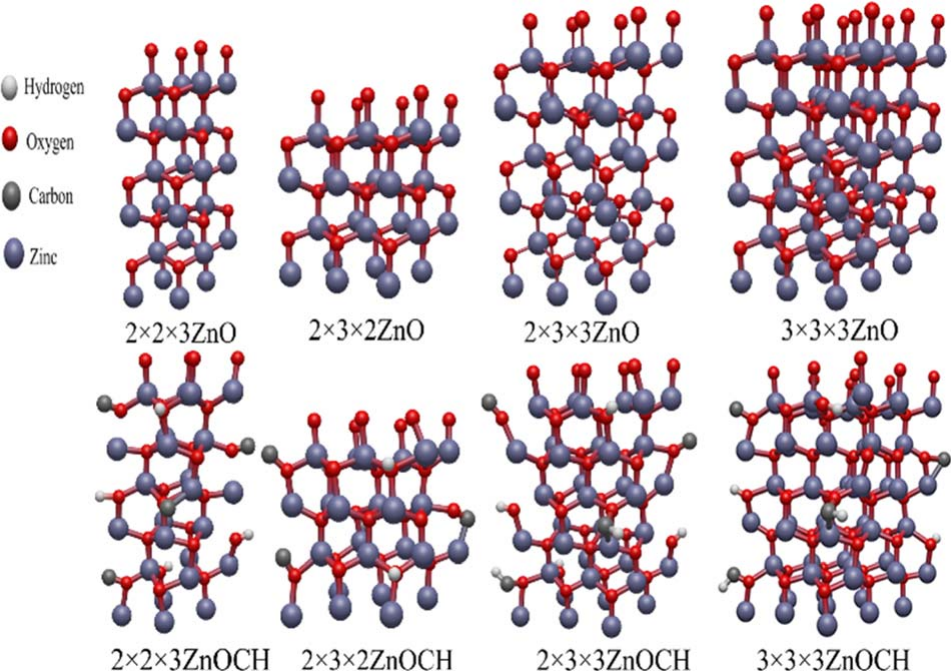
Optimized 2 × 2 × 3, 2 × 3 × 2, 2 × 3 × 3 and 3 × 3 × 3 supercells of ZnO and ZnOCH.

This research examined supercells including 2 × 2 × 3, 2 × 3 × 2, 2 × 3 × 3 and 3 × 3 × 3 of ZnO consisting of 24, 24, 36 and 54Zn atoms and 24, 24, 36 and 54O atoms, respectively (Fig. 1). To analyze the presence and absence of C=O, C–O, CH, and OH bands, four carbon atoms and four hydrogen atoms were considered, along with their effects on material properties such as defect formation energy and ESR spectra, in 2 × 2 × 3, 2 × 3 × 2, 2 × 3 × 3, and 3 × 3 × 3 supercells (Fig. 1). The mentioned supercells were optimized by calculating Density Functional Theory (DFT) through the PBE functional, DZVP Gaussian basis set, and Gaussian and Plane Waves (GPW) methods, in addition to GTH pseudopotentials, using the CP2K software package.^33^ The energy cut off was chosen to be 320 Ry.

The electronic structures and parameters of ESR as g-tensor components and hyperfine coupling constants (HFCCs) were then measured by applying the optimized structures in the QUANTUM ESPRESSO software.^34^ The spin-polarized, gradient-corrected PBE functional was employed in the DFT framework.^35^ A series of energy cutoffs—20, 30, 40, and 50 Ry—were tested for the auxiliary plane-wave basis set, along with various *k*-point configurations, including a 1 × 1 × 1, 1 × 2 × 2, 2 × 2 × 2, 2 × 3 × 3, 3 × 3 × 3 and 3 × 4 × 4 Monkhorst–Pack grid, until convergence in the energy band gap was achieved. To accurately describe the electronic structures, the DFT+U method was employed, applying the Hubbard U correction to the Zn-3d and O-2p states. Different values for Up(O) and Ud(Zn) were explored within the ranges of 0–12 eV and 8–12 eV, respectively, to ensure precise calculations of energy band gap. It was determined that an energy cutoff of 40 Ry, a *k*-point grid of 2 × 3 × 3 Monkhorst–Pack, Up(O) of 11 eV, Ud(Zn) of 10 eV, and a charge density of 320 provided optimal convergence. Finally, the formation energy and ESR parameters were calculated using the GW and Gauge-Including Projector Augmented Wave (GIPAW) method, respectively.^34^

## Results and discussion

In order to obtain more information about the structure of prepared zinc oxide nanoparticles and their effect along with the calcination temperature after the synthesis of nanoparticles (400, 500 and 600 °C) various structural investigations were carried out.

The chemical nature of zinc oxide samples was investigated by FT-IR spectroscopy at room temperature and more information about the role of calcination process in their formation mechanism was obtained. As shown in Fig. S1,† the bands at 445 and 492 cm^−1^ became sharper and cleaner after calcination of ZnO at 500 °C, which confirms the formation of Zn–O stretching vibration bond. The overlap of the mentioned bands at temperatures of 400 and 600 °C shows that the ZnO crystal network is completely formed at 500 °C. These two bands correspond to the hexagonal structure of ZnO. The bands at 1176 and 1271 cm^−1^ indicate the Lewis acidity caused by the C=C stretching vibration, which is caused by the presence of a small percentage of organic components in the samples. Also, the present bands at 1399, 1455 and 1617 cm^−1^ show the asymmetric stretching vibrations of Brønsted C=O acidity sites, CH_2_ bending and C–O symmetric stretching respectively, which are caused by chelating and cross-linking factors such as polyvinyl pyrrolidone and diethylene glycol. Carbon impurities can be found in the final samples, if the calcination energy is less than the amount of energy required for the growth of inorganic nanoparticles. Also, the vibrations of 2349 and 2413 cm^−1^, which belong to O–C–O molecules, shifted to higher wavelengths and disappeared along with the heating of the samples. The bands recorded in the region of 2924 cm^−1^ in ZnO samples are related to the asymmetric stretching vibrations of CH among the impurities, they have weakened and sometimes disappeared by applying the heating process. Finally, the broad band in the region of 3400 and 3545 cm^−1^, which indicates the presence of stretching vibrations of surface hydroxyl groups and stretching vibrations of water or moisture absorbed from the environment, shows pure ZnO based on calcination temperatures and ZnO pure nanoparticles show a decrease in both intensity and resolution in this band from 400 to 600 °C.

The formation and morphology of the synthesized nanoparticles were carried out under SEM magnification. The role of calcination temperature is shown in the corresponding micrographs in Fig. S2.† As shown in Fig. S2,† increasing temperature resulted in irregular shapes nanoparticles. In addition, homogeneity and small size distribution can be observed after calcination. The presence of some larger particles in the micrographs can be attributed to the clumping and overlapping of smaller particles during the synthesis process.

**Fig. 2:**
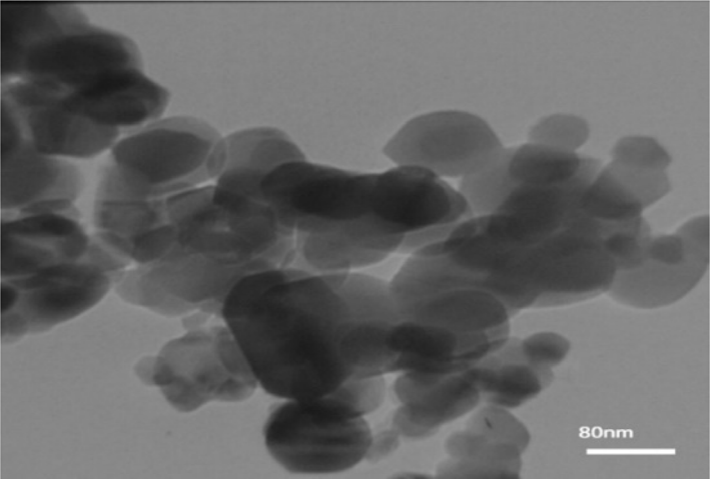
TEM micrographs of ZnO nanoparticles calcined at 500 °C.

TEM micrographs of ZnO at 500 °C is shown in Fig. 2. The micrograph confirms the nanometer-scale grain size of the sample, as well as the irregular shapes morphology. This article proves the polycrystalline nature of nanoparticles. In addition, the surfaces, edges and smooth and homogeneous structure of the nanoparticles are clear in the image, which confirms the good quality of the prepared nanostructures and provides an almost uniform distribution of dark and light areas. Size and shape studies confirm that nanocrystalline structures have been successfully obtained after the calcination process, which is consistent with the SEM microscopic result.

**Fig. 3:**
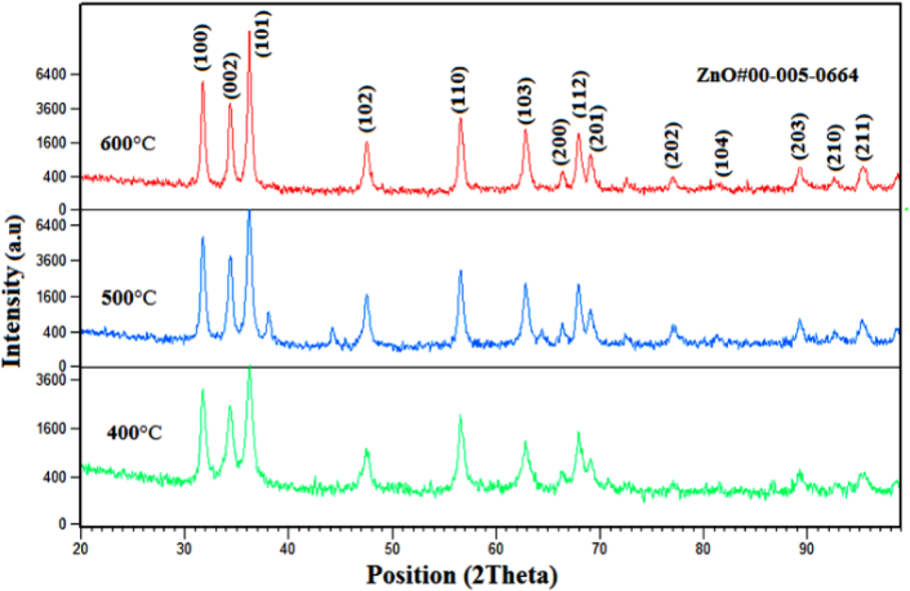
XRD patterns of ZnO nanoparticles calcined at 400, 500 and 600 °C.

In order to investigate the effect of temperature on the structural properties of ZnO nanoparticles, calcination was performed at temperatures of 400, 500 and 600 °C for 2 hours. Fig. 3 shows the X-ray diffraction patterns of ZnO nanoparticles calcined at different temperatures.

The phase identification obtained from XRD analysis shows that the ZnO phase is at the angles of 31.7263, 43.3185, 36.2672, 43.7376, 56.5757, 62.8713, 66.4008, 67.9343, 69.1294, 77.1440, 81.2778, 89.1972, 92.6086 and 95.2282 have pages (100), (002), (101), (102), (110), (103), (200), (112), (201), (202), (104), (203), (210) and (211) respectively, which has a hexagonal lattice appears. This phase is fully compatible with the standard card number 005-0664.

From the XRD data, the particle size of the prepared crystals was estimated by the Debye–Sherer equation.

The crystal size of prepared ZnO nanoparticles was calculated using the average of three long peaks at the angles of 31.7263°, 43.3185° and 36.2672° at temperatures of 400, 500 and 600 °C and was about 32, 34 and 37 nm respectively.

**Fig. 4:**
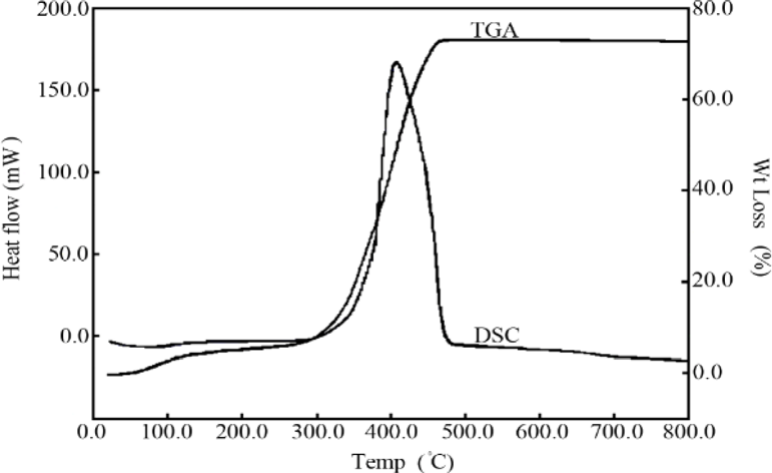
DSC and TGA curves of ZnO nanoparticles.

In order to explain the thermal behaviour of the prepared zinc oxide nanoparticles, their thermal analysis was investigated by TGA/DSC. As shown in Fig. 4, the total weight loss at 800 °C is about 69.5%. The weight loss from 40 to 110° is about 3.5% which accompanied with endothermic peaks in its DSC curves. The weight loss in this temperature range is primarily associated with the elimination of physically adsorbed water or solvent. The zinc oxide nanoparticles, also show about 66% weight loss in the 280–480 °C temperature range along with exothermic peaks in their DSC curves. These peaks are associated with the combustion of organic materials cross linking. The small exothermic peak observed at 650 °C in the DSC thermogram without weight loss in TGA can be attributed to the formation of the crystalline phase of zinc oxide.

**Fig. 5:**
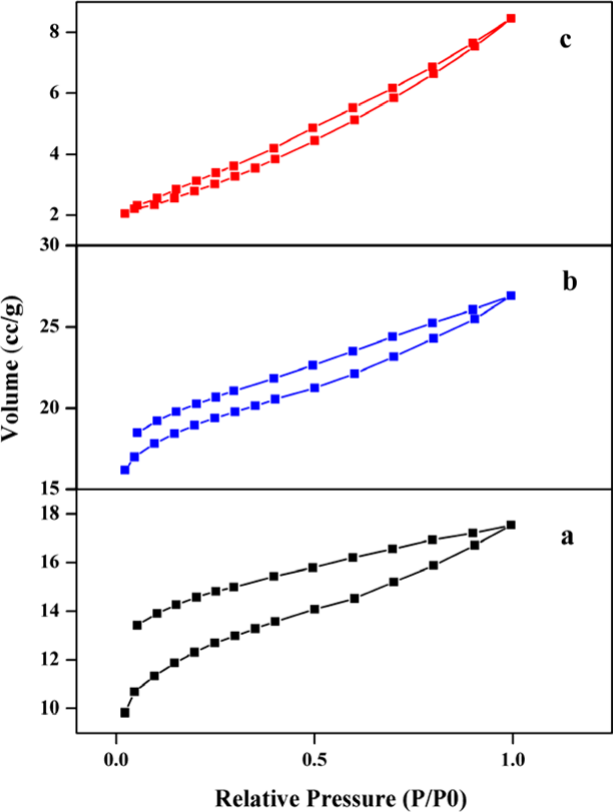
N_2_ adsorption–desorption isotherms of ZnO nanoparticles calcined at (a) 400, (b) 500 and (c) 600 °C.

The N_2_ adsorption–desorption isotherm at 77 K measured for ZnO nanoparticles after calcination at 400, 500 and 600 °C. Fig. 5 displays the isotherms of the three samples.

Nitrogen adsorption/desorption analyses were performed in order to investigate the textural properties of the resulted compounds. Based on the pore distribution, it is assumed that the isotherm plot is close to type IV and the pores are close to meso species on the basis of IUPAC definition. The calculation of surface area and pore size distribution was obtained based on the BET and BJH methods.^36^

The surface area, pore volume, and pore diameter were determined as 32.16 m^2^ g^−1^, 0.02 cm^3^ g^−1^ and 1.17 nm for ZnO nanoparticles after calcination at 400 °C and 35.43 m^2^ g^−1^, 0.03 cm^3^ g^−1^ and 1.20 nm for ZnO nanoparticles after calcination at 500 °C and 10.01 m^2^ g^−1^, 0.01 cm^3^ g^−1^ and 3.08 nm for ZnO nanoparticles after calcination at 600 °C, respectively (Table 1). The surface areas are in the range of 10–35 m^2^ g^−1^ and among all samples, ZnO-500 has the highest surface area, pore volume and pore size, and ZnO-600 has the lowest surface area. The absorption capacity in the samples increases by raising the temperature to 500 °C, because the crystallization process takes place.

**Table 1:** Physisorption results of ZnO nanoparticles^a^

ZnO (°C)	*S*_BET_ (m^2^ g^−1^)	TPV (ml g^−1^)	APD (nm)
400	32.16	0.02	1.17
500	35.43	0.03	1.20
600	10.01	0.01	3.08

a (TPV) Total Pore Volume, (APD) Average Pore Diameter.

**Fig. 6:**
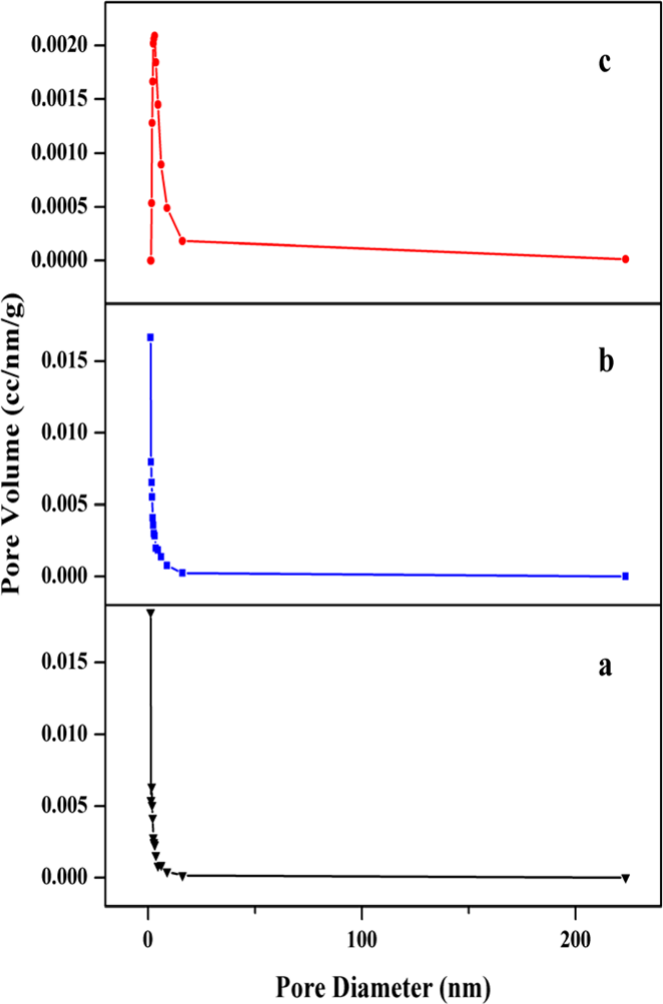
Pore size distribution of ZnO nanoparticles calcined at (a) 400, (b) 500 and (c) 600 °C.

After that, it decreases at 600 °C, because the size of the crystals has increased, which leads to less porosity and space between grain boundaries. In fact, this pattern can be related to the spaces between the grains in the samples. The number of adsorbed and desorbed molecules at low relative pressure (*P*/*P*_0_) (about 0.98) is used to check the specific surface area by BET and BJH analyses. From the results of the BET and BJH graphs shown in Fig. 6, it can be seen that a major fraction of the pore diameter in all samples is less than 5 nm.

As shown in the figures, the heating process shows an increase in the calculated surface area values of the BET and BJH analyses from 400 to 500 °C, where crystallization of the system occurs. While this parameter drops drastically for the heated sample at 600 degrees. Although at 600 degrees, the samples are completely crystalline, but increasing the temperature reduces the interfacial space through further growth of crystals, resulting in a lower surface-to-volume ratio.

**Fig. 7:**
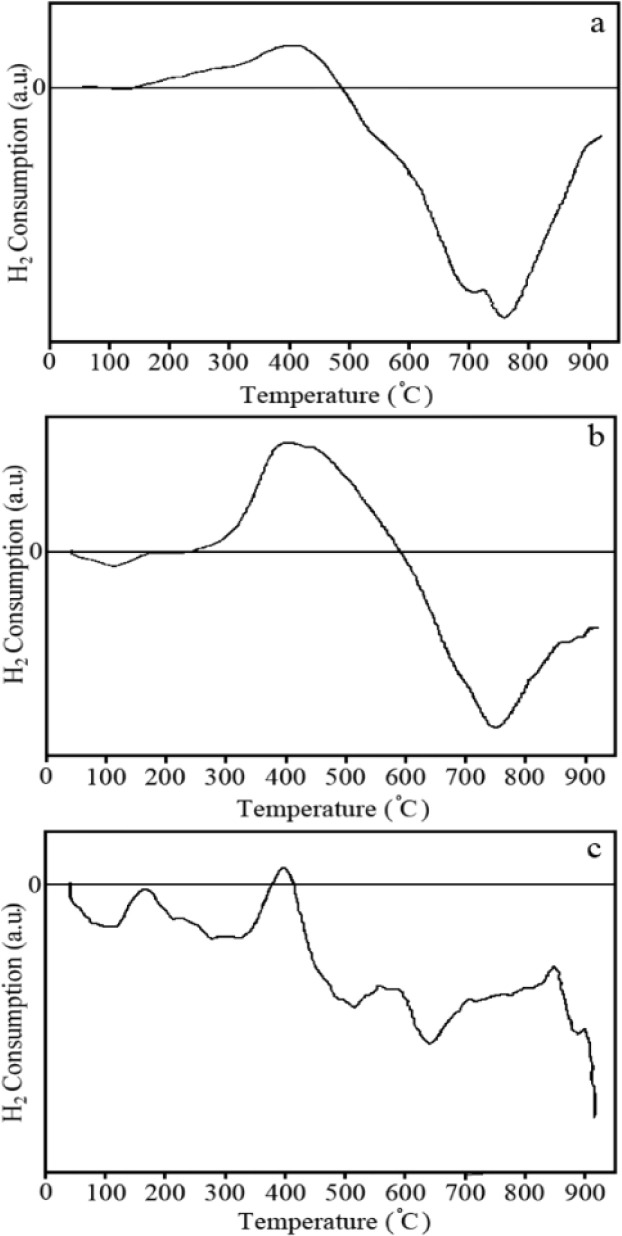
TPR profiles of ZnO nanoparticles calcined at (a) 400, (b) 500 and (c) 600 °C.

Reducibility of the prepared nanoparticles helps to better understand the structural features, especially the surface features. This behaviour was investigated by reduction reaction with hydrogen gas and the results of H_2_-TPR analysis profiles (Fig. 7).

Partial reduction of both surface and bulk in the ZnO structure can be observed by TPR diagrams of samples calcined at different temperatures. These observations relate the position of TPR peaks to the crystal size. With the growth of ZnO crystals by heating until reaching the full crystal lattice at 500 °C, the surface-to-volume ratio decreases and more mass atoms become available. Hence, higher temperatures are required for their location of the reduction peaks (around 100 °C) which regeneration. The difference in the intensity and appeared as a wide band overlapping between ZnO nanoparticles calcined at 400 and 500 °C is proof of this.

Hydrogen consumption for ZnO nanoparticles calcined at 400 °C appears as an overlapping band consisting of small shoulders at 220 and 280 °C and the main peak at 400 °C. Partial reduction at relatively low temperatures can be attributed to hydrogen absorbed both on the surface and in the mass of ZnO nanoparticles, which is carried out as the following reaction:ZnO + H_2_ → HZn–OH

The shoulders become weaker for calcined ZnO nanoparticles at 500 °C, but leave the main peak centered at 400 °C.

**Fig. 8:**
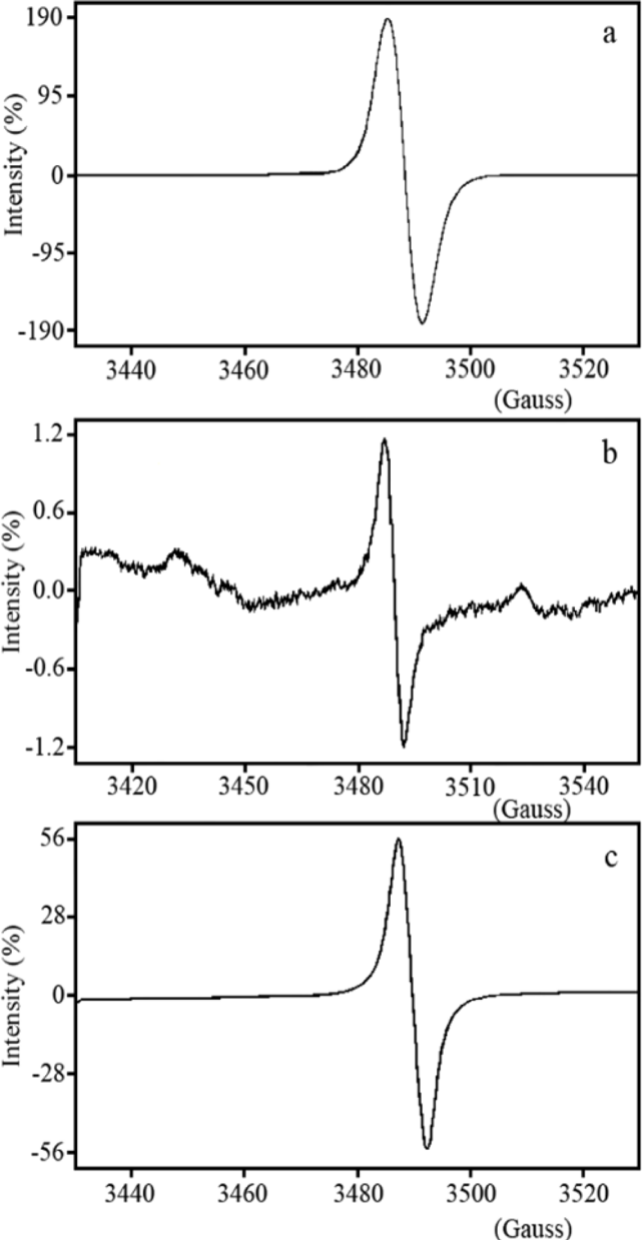
ESR signals of ZnO nanoparticles calcined at (a) 400, (b) 500 and (c) 600 °C.

Oxidation and electron spin states of the synthesized nanoparticles were studied by electron spin resonance (ESR) spectroscopy (Fig. 8). ZnO bulk powder on the micrometer scale is antimagnetic and does not exhibit any activity in the presence of a magnetic field.^37^ But experimental research has revealed that stability fluctuations in ZnO profiles can be obtained, where the conditions of the synthesis method and optimization are decisive for various magnetic properties. Here, polycrystalline ZnO nanoparticles with randomly oriented planes show broad isotropic and Gaussian-shaped ESR spectra at ambient temperature with constant peak-to-peak splitting (Δ*H*_pp_). The relative intensities of the ESR signals for the samples obtained in the heating process at 400, 500 and 600 °C are about 190.0, 1.20 and 56.0%, respectively (Fig. 8a–c). Fluctuation in ESR intensities in the presence of an applied magnetic field expresses the direct effect of the calcination process on the structure of ZnO nanoparticles.

The ZnO sample calcined at 400 °C is not yet fully crystallized and has a large number of defects, including positions caused by charged oxygen vacancy and interstitial zinc species in its structure, both on the surface and in the bulk. The observed broad signal points to the combination of local magnetic fields in the ZnO system caused by both defects related to zinc and oxygen species with the external magnetic field. On the other hand, until now, a large number of researches have been conducted in the field of studying the electron spin resonance behaviour in the ZnO system, which relate the splitting to oxygen vacancy species with a negative charge (V_O_^−1^) or positive charge (V_O_^+1^).^38,39^

Oxygen vacancy may be present, but not persistent (Fig. 12a). Conversely oxygen vacancy capable of trapping electrons in the presence of an applied magnetic field, thus producing paramagnetic F centers.^40^ According to the results obtained from H_2_-TPR analysis, interstitial centers on (Zn^+1^) (Zn → Zn^+1^ + e^−^) and (Zn^−1^) (Zn + e^−^ → Zn^−1^) can originate from the ionization of charged oxygen vacancy species on the surface of nanoparticles, and hence they create paramagnetic resonances with total spin angular momentum (S) equal to 1.2. The fully stable ESR signal at room temperature leads to the presence of charged interstitial Zn and charged oxygen vacancy species sites, it was explained in theoretical section. By applying the heating process on the samples after synthesis and obtaining the crystal lattice for the ZnO sample calcined at 500 °C, the intensity of the ESR signal drops sharply. Electrons are more bound in this system and as the H_2_-TPR profile of this sample confirms, their availability leads to the reduction of Zn^+1^ and Zn^−1^ interstitial species to Zn. A further increase in ZnO crystal size with calcination at 600 °C led to a sharp drop in surface effects, and subsequently, defects related to charged zinc vacancy and charged interstitial oxygen.

### Theoretical results

As highlighted in the ESR analysis, the weak ferromagnetic ordering observed in the ZnO structure might be caused by the native defects and defect complexes. In this part of the study, the native defects in the ZnO and ZnOCH structure were identified by utilizing DFT within the formalism of GGA+U at 400, 500 and 600 °C.

First, the effects on the electronic structure of ZnO were accurately investigated by optimizing the geometrical structures of pure ZnO and those incorporated with C=O, C–O, CH, OH bands and native defects were predicted. Table 2 lists the Lattice parameters for unit cell of optimized 2 × 2 × 3, 2 × 3 × 2, 2 × 3 × 3 and 3 × 3 × 3 supercells of ZnO and ZnOCH. Lattice parameters of a and *c* respectively calculated to be 3.27 Å and 5.22 Å for the optimized 2 × 3 × 3 and 3 × 3 × 3 supercells of pure ZnO. Low deviation was found between the calculated and experimental values,^41,42^ demonstrating the model's reliability and validity. The lattice parameters increased after the introduction of C=O, C–O, CH, OH bands.

**Table 2:** Lattice parameters for unit cell of optimized 2 × 2 × 3, 2 × 3 × 2, 2 × 3 × 3 and 3 × 3 × 3 supercells of ZnO and ZnOCH

	ZnO		ZnOCH	
	*a* (Å)	*c* (Å)	*a* (Å)	*c* (Å)
2 × 2 × 3	3.37	5.16	3.53	5.36
2 × 3 × 2	3.41	5.23	3.62	5.41
2 × 3 × 3	3.27	5.22	3.34	5.30
3 × 3 × 3	3.26	5.22	3.34	5.23

In practice, the supercell sizes required to achieve absolute convergence would be prohibitively large for feasible calculations. We performed band structure calculations for supercells that included 2 × 2 × 3, 2 × 3 × 2, 2 × 3 × 3 and 3 × 3 × 3 of ZnO (see Fig. S3†). Also, accurate determination of the energy band gap of ZnO is critically dependent on ensuring that all calculations converged across a wide range of variables, including energy cutoff, *k*-point selection, Hubbard U corrections applied to the Zn-3d and O-2p states, and charge density (see Fig. S4–S7†). Our evaluations indicated that for 2 × 3 × 3 supercell with an energy cutoff of 40 Ry, a charge density of 320 Ry, a *k*-point grid of 2 × 3 × 3 (Monkhorst–Pack), Up(O) of 11 eV, and Ud (Zn) of 10 eV were necessary for accurate determination of the energy band gap.

**Fig. 9:**
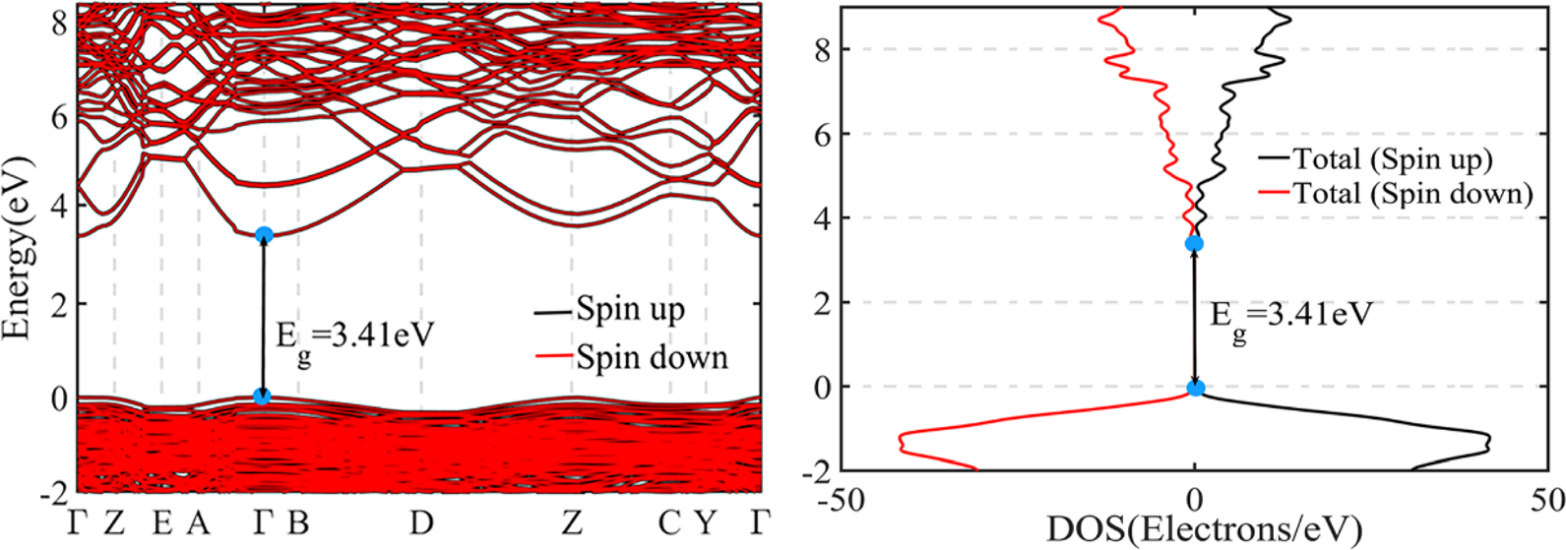
The electronic band structures, TDOS of pure ZnO.

These structures, together with the spin-polarized, orbital- the projected Density of States (DOS) are presented in Fig. 9. The semiconductor of pure ZnO with its valence band at the bottom and top of the conduction bands located at the *Γ* point of the Brillouin zone and the resulted direct band gap is shown in Fig. 9. Its optical band gap was calculated to be 3.41 eV, which has good agreement with its experimental value (3.37 eV).^41,42^

The formation energies were computed for all the defect types mentioned so as to determine the thermodynamic stability and identify the most abundant native defects and defect complexes. These energies for native defects could be calculated in different charge states (0, +1, and −1) as follows:^43^1
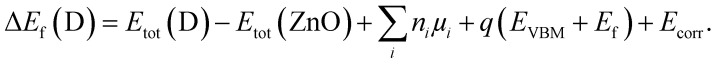
where *E*_tot_(ZnO) and *E*_tot_(D) stand for the total energy of the pure ZnO and the defective ZnO supercell, respectively; *n*_i_ shows the number of atoms of type *i* added to the supercell or removed from it with negative and positive values, respectively; *μ*_*i*_ is the chemical potential of the atomic species *i* (Zn or O); *q* represents the defect charge; *E*_VBM_ is the valence band maximum for pure ZnO and *E*_F_ demonstrates the Fermi energy. Finally, *E*_corr_ represents a sum of relevant correction terms. In our case, it addressed the interaction between the defect and its spurious periodic images, specifically correcting for the unwanted coulombic interactions between a charged defect and its nearest neighboring images due to periodic boundary conditions. Since the formation energy was calculated using periodically repeated finite-sized supercells, artificial long-range elastic and electrostatic interactions could arise between the periodic defect images. If the supercells are not sufficiently large in all dimensions, the Coulomb interactions between the defect charge and its nearest periodic images will not diminish, leading to a spurious contribution to the total energy of the defective system. However, when sufficiently large supercells are employed, the interactions between the defect and its spurious periodic images, as well as with the jellium background, become negligible.^44^

The chemical potential is influenced by the experimental conditions, under which the material is synthesized. To determine these quantities, we used the relationship *μ*_Zn_ + *μ*_O_ = *μ*_ZnO_, assuming both species were in thermal equilibrium with ZnO. Additionally, the chemical potentials had to adhere to the boundary conditions *μ*_O_ ≤ 1/2*μ*_O_2__ and *μ*_Zn_ ≤ *μ*_Zn_ (bulk). Under specific growth conditions, we considered oxygen-rich scenarios where *μ*_O_ = 1/2*μ*_O_2__ or zinc-rich scenarios where *μ*_Zn_ = (bulk). In oxygen-rich conditions, *μ*_Zn_ = *E*_ZnO_ (bulk) − 1/2*E*_O_2__ and in zinc-rich conditions, *μ*_O_ = *E*_ZnO_ (bulk) − 1/2*E*_Zn_ (bulk). Here, the *E*_ZnO_ (bulk), *E*_Zn_ (bulk), and *E*_O_2__ represented the total energies of bulk ZnO, Zn, and gas-phase O_2_ molecules, respectively. For the gas-phase O_2_ molecules, we simulated two oxygen atoms at a distance of 1.2 Å within artificial periodic conditions in a cubic box with a side length of 10 Å. The value of the chemical potential *μ*_O_ was derived from the total energy of an O_2_ molecule. The calculated chemical potential of the atomic species zinc and oxygen under three conditions and total energy of the ZnO and ZnOCH with incorporating native defects listed in Tables S1–S9.†

**Fig. 10:**
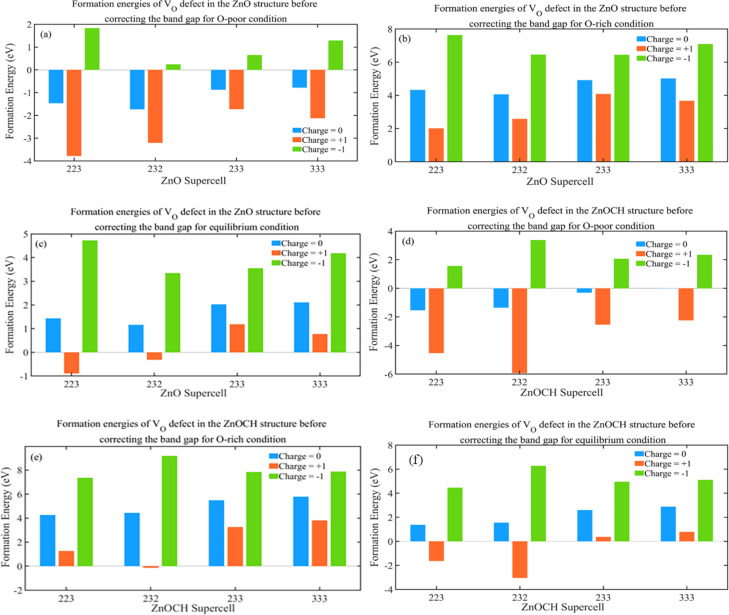
Formation energies of V_O_ defect in 2 × 2 × 3, 2 × 3 × 2, 2 × 3 × 3 and 3 × 3 × 3 supercells for ZnO (a–c) and ZnOCH (d–f) structures before correcting the band gap (values for O-poor, O-rich and equilibrium conditions are presented at *E*_F_ = 0 eV).

The growth environment could affect the formation energy during the experimental process of preparation. Low and high flow rates of oxygen at low and high temperatures were regarded as oxygen-poor and -rich conditions, respectively. Now, let us examine the effect of supercell size on the formation energy. To study V_O_ defects, we constructed supercells of sizes 2 × 2 × 3, 2 × 3 × 2, 2 × 3 × 3, and 3 × 3 × 3, each containing a V_O_ defect (Fig. S8†), and performed complete structural relaxations. The formation energies of the V_O_ defects were measured under three conditions at *E*_F_ = 0 eV defect in 2 × 2 × 3, 2 × 3 × 2, 2 × 3 × 3 and 3 × 3 × 3 supercells for ZnO and ZnOCH structures before correcting the band gap and showed in Fig. 10. As shown, the formation energies for V_O_ defects in 2 × 3 × 3 and 3 × 3 × 3 supercells display only a slight difference. The highest value, approximately 0.64, corresponds to the −1-charge state under oxygen-rich and equilibrium conditions. The Coulomb interactions between the defect charge and its nearest periodic images will insignificant. Consequently, 2 × 3 × 3 supercell was employed to calculate the formation energies of other defects.

**Fig. 11:**
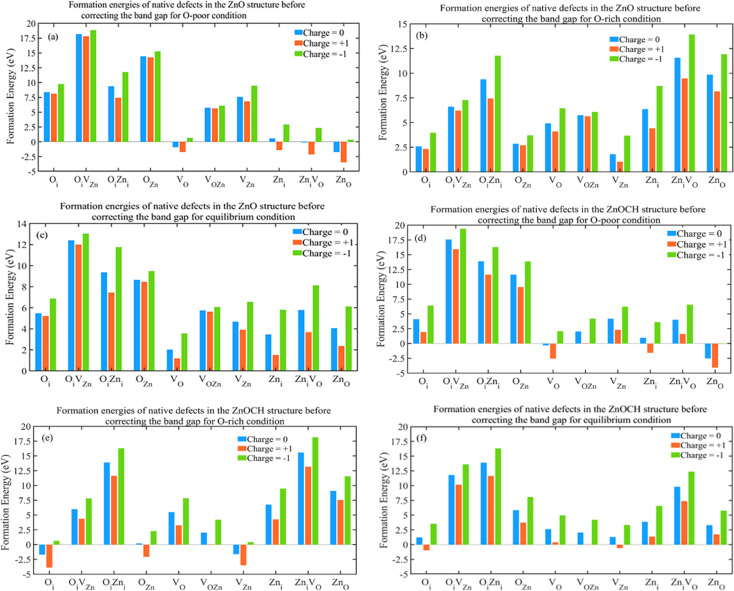
Formation energies of native defects in 2 × 3 × 3 supercell for ZnO (a–c) and ZnOCH (d–f) structures before correcting the band gap (values for O-poor, O-rich and equilibrium conditions are presented at *E*_F_ = 0 eV).

The supercells of ZnO and ZnOCH with incorporating native defects are depicted in Fig. S9.† The formation energies of the native defects were measured under both conditions at *E*_F_ = 0 eV and showed in Fig. 11.

Lower formation energies indicated a higher likelihood of defect occurrence. The following key points could be made with regard to the calculated results: under oxygen-poor conditions (400 °C) and *E*_F_ = 0 eV, Zn_O_(Zn_O_), V_O_(V_O_), Zn_i_V_O_(Zn_i_V_O_) and Zn_i_(V_OZn_) for ZnO (ZnOCH) structure had the lowest formation energies, showing to be the most dominant defects, respectively. Under equilibrium conditions (500 °C) and *E*_F_ = 0 eV, V_O_(O_i_) for ZnO (ZnOCH) structure had the lowest formation energies, showing to be the most dominant defect. Similarly, under oxygen-rich conditions (600 °C) and *E*_F_ = 0 eV, V_Zn_(O_i_), O_i_(V_Zn_) and O_Zn_(O_Zn_ and V_OZn_) for ZnO (ZnOCH) structure had the lowest formation energies, showing to be the most dominant defect, respectively. For three conditions, the formation energies of the O_i_V_Zn_(O_i_V_Zn_), O_i_Zn_i_(O_i_Zn_i_) and V_OZn_(Zn_i_V_O_) defects in different charge states were too high, indicating that they were present in very low abundances.

Energy levels are often introduced by defects within the band gaps of semiconductors corresponding to transitions between varied charge states of the same defects. These transition levels, *ε*(*q*/*q*′), defined as the position of Fermi level with equal formation energies of the charge states of *q* and *q*′ can be derived using the following expression:^45^2
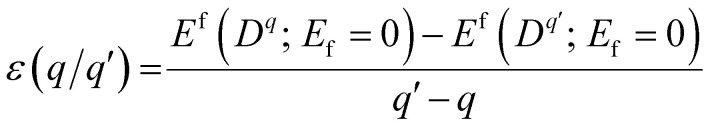


**Fig. 12:**
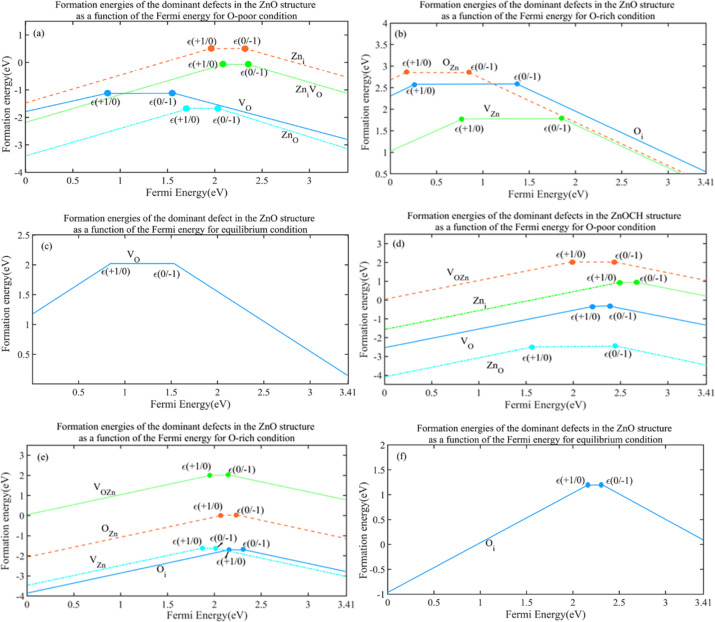
Calculated formation energies of the most dominant defects in their charged states under oxygen-poor, oxygen-rich and equilibrium conditions for ZnO (a–c) and ZnOCH (d–f) structures as a function of the Fermi energy, respectively.

Considering the Fermi level at the maximum valence band, the formation energy of a *D* defect in the charge state of *q* is denoted as *E*^f^ (*D*^*q*^; *E*_f_ = 0). The significance of the transition level of *ε*(*q*/*q*′) is that the charge states of *q* and *q*′ are stable when the Fermi-level positions are below and above that level, respectively. Fig. 12 plots the formation energies of the most dominant defects in their charged states under oxygen-poor, oxygen-rich and equilibrium conditions as a function of the Fermi energy. As the figure shows, defects are not persistent in a neutral charge state for ZnOCH structure.

Furthermore, we simulated the ESR spectra of the native defects for verification. The unpaired electrons localized on the paramagnetic species, which might be intrinsic to the material or triggered by the defects, were detected through ESR spectroscopy. An ESR spectrum arises from the interaction of an external magnetic field with an unpaired electron spin, as well as the interaction with neighbouring nuclear spins known as the interaction of HFCCs. The resonance field position was determined in the simulated ESR spectrum by calculating ESR parameters like the *g*-factor, HFCCs, and basic parameters of magnetic field sweep range and microwave frequency as follows:^46^3
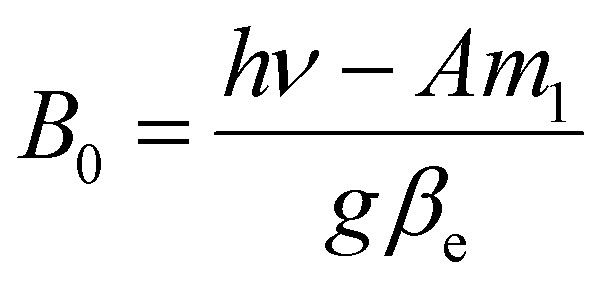
where *m*_1_, *β*_e_, and *B*_0_ represent the quantum number of nuclear spin, Bohr magneton, and magnetic field strength in mT, respectively.

However, since only the isotopes of oxygen-17 and zinc-67 possessed a nuclear spin and HFCC and their natural abundances are negligible and hydrogen atoms, which possessed a high-abundance isotope with a nuclear spin, exhibited a very small HFCC therefore the HFCC had a negligible effect on the ESR spectrum and the resonance field position.

**Fig. 13:**
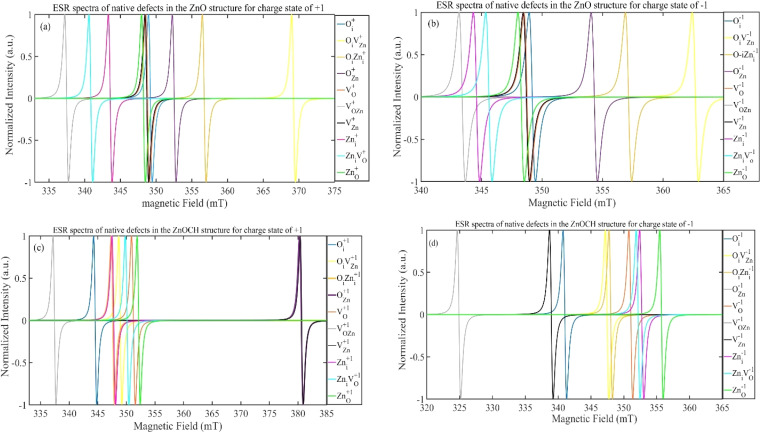
The simulated ESR spectra of the native defects in the ZnO (a and b) and ZnOCH(c and d) structures for charge states of +1 and −1 in room temperature. Microwave frequency *ν* = 9.77 GHz.

Hence, the centers of the ESR spectrum were primarily delineated by the electronic *g*-value. It is important to note that charged defect states are paramagnetic, while neutral defect states are diamagnetic and do not contribute to the ESR spectrum. The simulated EPR spectra for the native defects in the charge states of +1 and −1 are portrayed in Fig. 13. By comparing the positions of resonance fields in the simulated ESR spectrum with those in the experimental spectrum, one could infer the presence or absence of defects. However, the overlap of ESR spectra resulting from different defects made it impossible to determine defect concentrations accurately.

Comparison of the calculated resonance field positions for native defects in the ZnO structure (Fig. 13) with the experimental ESR spectra in Fig. 8 indicated that the concentrations of the **O**_**i**_**V**_**Zn**_^+1(−1)^, **O**_**i**_**Zn**_**i**_^+1(−1)^ and **V**_**OZn**_^+1(−1)^ defects were very low or practically zero that had the height formation energy in three conditions. The high intensity of the ESR spectrum at 348.5 mT for the sample prepared at 400 °C suggested a high concentration of **Zn**_**O**_^+1(−1)^ and **V**_**O**_^+1(−1)^ that had the lowest formation energies at 400 °C. The decrease in the intensity and width of the ESR spectrum at 348.5 mT for the sample prepared at 500 °C indicated that the concentrations of the **V**_**O**_^+1(−1)^ defect had decreased that had the lowest formation energies at 500 °C. The ESR spectrum at 348.5 mT for the sample prepared at 600 °C suggested a relatively high concentration of **V**_**Zn**_^+1(−1)^ and **O**_**i**_^+1(−1)^ defects that had the lowest formation energies at 600 °C and low concentration of **O**_**Zn**_^+1(−1)^ defect that had approximately low concentration in 600 °C. The ESR spectra indicated that the lowest defect concentration occurred at 500 °C, suggesting that this temperature represented the equilibrium state for synthesizing zinc oxide.

A comparison of the calculated resonance field positions for native defects in the ZnOCH structure (Fig. 13) with the experimental ESR spectra in Fig. 8 indicated that the concentrations of the defects **O**_**Zn**_^+1(−1)^, **V**_**O**_^+1(−1)^, **V**_**OZn**_^+1(−1)^, **V**_**Zn**_^+1(−1)^, **Zn**_**i**_**V**_**O**_^+1(−1)^, **Zn**_**O**_^+1(−1)^ were very low or practically zero, which had low formation energy under at least one of the growth environment conditions. In contrast, there was a relatively high concentration of the **Zn**_**i**_^+1(−1)^ and **O**_**i**_**Zn**_**i**_^+1(−1)^ defects, with the **O**_**i**_**Zn**_**i**_^+1(−1)^ defect having the hight formation energy in three conditions. Additionally, the concentration of **O**_**i**_**Vzn**^+1(−1)^ and **O**_**i**_^+1(−1)^ defects were relatively low, with the **O**_**i**_^+1(−1)^ defect exhibiting very low formation energy in o-rich condition.

A comparison of the resonance field positions in the simulated ESR spectrum for ZnOCH with the experimental spectrum revealed that defects with lower formation energies appeared at field positions that did not align with the experimental field positions. In contrast, defects with higher formation energies coincided with the experimental field positions. This inconsistency between formation energy and field position suggested that C=O, C–O, CH, OH bands were negligible and could be disregarded in the ZnO nanoparticles.

## Conclusions

In conclusion, the study on zinc oxide nanoparticles (ZnO-NPs) revealed valuable insights into their formation, structural properties, and morphology. The FT-IR spectroscopy analysis confirmed the presence of Zn–O stretching vibration bonds, while SEM and TEM imaging revealed irregular shapes with nanometer-scale grain sizes. The successful formation nanocrystalline structures after calcination was further supported by XRD patterns. Moving forward, addressing any impurities, optimizing calcination temperatures and exploring application-specific behaviours will deepen our understanding of ZnO-NPs and improve their practical applications across various fields.

Additionally, the investigation into ZnO-NPs highlighted intriguing behaviours related to calcination temperature. The reduction peaks observed in TPR diagrams indicated significant changes in both surface and bulk properties, while ESR spectroscopy provided insights into electron spin states and magnetic behaviour. The interplay between defects, crystallization, and stability emphasizes the complexity of ZnO-NPs and their potential for diverse applications.

The results of the formation energy calculations and ESR spectra simulations showed that during the ZnO preparation process, the growth environment influences the native defects that occur. The calculated formation energies and simulated ESR spectra indicated that under oxygen-poor conditions (400 °C), the defects **Zn**_**O**_, **V**_**O**_, **Zn**_**i**_**V**_**O**_ and **Zn**_**i**_ were more likely to occur, while V_**Zn**_, **O**_**i**_ and **O**_**Zn**_ were more probable under oxygen-rich conditions (600 °C). Under equilibrium conditions (500 °C), V_**O**_ defect, with low concentrations, was the dominant defect. Therefore, the formation energies and ESR spectra of defects suggest that the optimal conditions for preparing zinc oxide nanoparticle occurred in the 500 °C. Finally, the inconsistency between calculated of formation energy and the field position suggested that the C=O, C–O, CH, and OH bands were negligible and could be disregarded in the ZnO nanoparticles.

This study on ZnO nanoparticles reviews various synthesis methods, focusing on the combination of sol–gel and hydrothermal techniques, which produce high-purity nanoparticles with controlled morphology while analysing the impact of calcination temperature on defect formation. It effectively utilizes Density Functional Theory (DFT) to investigate native defects examining their influence on electronic and magnetic properties in oxygen-rich and oxygen-poor environments. The research reveals insights into the weak ferromagnetic ordering attributed to defects and emphasizes the critical role of calcination temperature in determining structural and electronic properties, linking it to defect formations. By highlighting the applications of ZnO nanoparticles in areas like photocatalysis, sensors, and spintronics, the study offers a fresh perspective on optimizing their performance through tailored synthesis and defect engineering. Overall, it advances the understanding of nanomaterial properties and their implications for various technological applications.

## Data availability

I confirm that all the data mentioned in the manuscript is available upon request and will be made available to the reviewers and readers upon acceptance for publication.

## Conflicts of interest

There are no conflicts to declare.

## Supplementary Material

RA-014-D4RA04252B-s001

## References

[cit1] Afolabi R. O. (2024). J. Mol. Liq..

[cit2] AbdelHamid A. A., Garcia A. M., Lee S. S., Ying J. Y. (2024). Nano Energy.

[cit3] Hu Y., Chen D., Hei H., Yu Sh., Gao J., Ma Y., Zheng K., Wu Y., Zhou B. (2024). Ceram. Int..

[cit4] Jabeen H., Shaukat S., Abd Ur Rahman H. M. (2024). J. Mol. Liq..

[cit5] Vithalani P., Bhatt N. (2024). Environ. Ecol..

[cit6] Jeevanandam J., Barhoum A., Chan Y. S., Dufresne A., Danquah M. K. (2018). J. Nanotechnol..

[cit7] Red A. T., Park J. Y., Park Y. T. (2024). J. Funct. Biomater..

[cit8] Jia T., Wang W., Long F., Fu Z., Wang H., Zhang Q. (2009). J. Mater. Sci. Eng. B.

[cit9] Motla A., Kumaravelu Th. A., Dong Ch. L., Chen Ch. L., Asokan K., Annapoorni S. (2024). J. Mater. Sci.: Mater. Electron..

[cit10] Slimani Y., Caliskan S., Khan F. A., Baykal A., Almessiere M. A. (2024). Opt. Mater..

[cit11] Shi J., Zhang J., Yang L., Qu M., Qi D. Ch., Zhang K. H. L. (2021). Adv. Mater..

[cit12] Wei A., Pan L., Huang W. (2011). Mater. Sci. Eng., B.

[cit13] Sathiyaseelan A., Naveen K. V., Zhang X., Han K., Wang M. H. (2023). Coord. Chem. Rev..

[cit14] Etafo N. O., Bamidele M. O., Bamisaye A., Alli Y. A. (2024). J. Water Proc. Eng..

[cit15] Krieguer B., Marque S. R. A., Dorey S., Dupuy N., Girard F., Perier N. G., Kuntz F., Ludwig N. (2024). J. Appl. Polym. Sci..

[cit16] Jiang X., Boudreau M. D., Fu P. P., Yin J. (2021). J. Environ. Sci. Health, Part C: Environ. Carcinog. Ecotoxicol. Rev..

[cit17] Lei J., Liu W., Jin Y., Li B. (2022). Anal. Chem..

[cit18] Caliskan B., Caliskan A. C. (2021). Radiat. Phys. Chem..

[cit19] Parashar S. K. S., Murty B. S., Repp S., Weber S., Erdem E. (2012). J. Appl. Phys..

[cit20] Schifano R., Jakiela R., Galeckas A., Kopalko K., Herklotz F., Johansen K. M. H., Vines L. (2019). J. Appl. Phys..

[cit21] Rai H., Kondal N. (2022). Mater. Today.

[cit22] Cerrato E., Paganini M. C., Giamello E. (2020). J. Photochem. Photobiol., A.

[cit23] Buryi M., Remeš Z., Babin V., Novotný M., Vaněček V., Dragounová K. A., Mičová J., Landová L., Kučerková R., More-Chevalie J., Chertopalov S., Fitl P., Kmječ T. (2021). Appl. Surf. Sci..

[cit24] Lokesha H. S., Prinsloo A. R. E., Mohanty P., Sheppard C. J. (2021). J. Alloys Compd..

[cit25] Karagoz E., Altaf C. T., Yaman E., Yildirim I. D., Erdem E., Celebi C., Fidan M., Sankir M., Sankir N. D. (2023). J. Alloys Compd..

[cit26] Bedhouche F., Soualah A., Djouadi D., Ahouari H., Tayeb K. B. (2023). Water, Air, Soil Pollut..

[cit27] Navale Sh. C., Ravi V., Srinivas D., Mulla I. S., Gosavi S. W., Kulkarni S. K. (2008). Sens. Actuators, B.

[cit28] Rakhimkulov Sh., Absattorov D., Borikhonov B., Yakubov E. (2024). Res. J. Chem. Environ..

[cit29] Saleem Sh., Jameel M. H., Rehman A., Tahir M. B., Irshad M. I., Jiang Zh. Y., Malik R. Q., Hussain A. A., Rehman A., Jabbar A. H., Alzahrani A. Y., Salem M. A. (2022). J. Mater. Res. Technol..

[cit30] Mende L. S., Driscoll J. L. M. (2007). Mater. Today.

[cit31] Sun Y., Zhang W., Li Q., Liu H., Wang X. (2023). Adv. Sens. Energy Mater..

[cit32] Soni B., Makkar S., Biswas S. (2021). J. Alloys Compd..

[cit33] VandeVondele J., Krack M., Mohamed F., Parrinello M., Chassaing T., Hutter J. (2005). Comput. Phys. Commun..

[cit34] Paolo G., Baroni S., Bonini N., Calandra M., Car R., Cavazzoni C., Ceresoli D., Chiarotti G. L., Cococcioni M., Daboet I. (2009). J. Phys.: Condens.Matter.

[cit35] Perdew J. P., Burke K., Ernzerhof M. (1996). Phys. Rev. Lett..

[cit36] Ward A. J., Pujari A. A., Costanzo L., Masters A. F., Maschmeyer T. (2011). Catal. Today.

[cit37] Mo L., Wan A., Zheng X., Yeh C. T. (2009). Catal. Today.

[cit38] Reddy R. J., Kokila M. K., Nagabhushana H., Chakradhar R. P. S., Nagabhushana B. M. (2011). J. Alloys Compd..

[cit39] Vlasenko L. S. (2010). Appl. Magn. Reson..

[cit40] Mihalache V., Secu M., Negrila C., Bercu V., Mercioniu I., Leca A. (2020). J. Mater. Sci. Eng. B.

[cit41] Indrajith E., Bhojya H. S., Ranga V., Kirthan B. R. (2020). Chem. Data Collect..

[cit42] Toufiq A. M., t Hussain R., Shah A., Mahmood A., Rehman A., Khan A., Rahman Sh. (2021). Phys. B.

[cit43] Freysoldt C., Grabowski B., Hickel T., Neugebauer J., Kresse G., Janotti A., Walle Van de C. G. (2014). Rev. Mod. Phys..

[cit44] Laaksonen K., Ganchenkova M. G., Nieminen R. M. (2008). J. Condens Matter. Phys..

[cit45] Zagorac D., Doll K., Schön J. C., Jansen M. (2011). Phys. Rev. B: Condens. Matter Mater. Phys..

[cit46] Janbazi M., Basaadatet M. R., Sasani Ghamsari M., Ahmady Sh. (2023). Mol. Phys..

