# Supplementary material for: Effect of temperature on the structure, catalyst and magnetic properties of un-doped zinc oxide nanoparticles: experimental and DFT calculation
Source: RSC Adv. 2024 Sep 30;14(42):31153–64. doi: 10.1039/d4ra04252b (PMC11441191; doi:10.1039/d4ra04252b)
Supplement: RA-014-D4RA04252B-s001 [file RA-014-D4RA04252B-s001.pdf]

Supplementary Information (SI) for RSC Advances.
This journal is © The Royal Society of Chemistry 2024

Effect of temperature on the structure, catalyst and magnetic properties of un-doped Zinc Oxide Nanoparticles: Experimental and DFT calculation

Masoomeh Sharbatdaran*, Mehdi Janbazi
Physics and Accelerators School, Nuclear Sciences and Technology Research Institute, Karaj, Iran

Supporting information

Material and instrumentation

Zinc nitrate (Zn $(NO_3)_2$), polyvinyl pyrrolidone (PVP), diethylene glycol (DEG, 98%), ethanol, and NaOH were purchased from Merck company. All chemicals were used as received and were of chemical grade. Deionized water was used for the preparation of all the solutions.

Infrared spectra were recorded on an ATI Mattson instrument, using KBr pellets. X-ray diffraction (XRD) patterns were recorded on a Philips PW-1800 diffractometer with Cu Kα radiation. Thermal analysis was carried out on Rheometric Scientific STA-1500 with heating rate of 10°C min-1 in air atmosphere. The microscopic morphology and nanometric grain size of the prepared nanoparticles were recorded by scanning electron microscopy (SEM, Philips XL-30) and transmission electron microscopy (TEM, Philips CM 10 HT, 300 kV). Prior to SEM and TEM microscopy, powders were coated with gold/palladium film and a Cu-carbon grid, respectively. Surface areas, pore volume and pore size distributions were obtained from the N2 isotherms which were determined at 77 K using Quantachrome Nova 2200, Version 7.11 Analyzer. Surface area was calculated by using BET equation, and pore size distribution was calculated by the BJH method based on the desorption branch.

Temperature programmed reduction of hydrogen (H2-TPR) measurements were performed on 30 mg of ZnO nano powder placed in a U-shaped quartz reactor using a Nano SORD NS91 system (Sensiran co., Iran). Prior to H2-TPR run, the sample was degassed in a flow of 10 sccm Ar at 300°C for 1 h, and cooled down to room temperature under the same atmosphere. The sample was then reduced by 10 sccm of 5.0% H2/Ar mixture while the temperature was raised to 950 °C with a heating rate of 10 °C/min. The chemical state and radical species assessment were conducted via electron spin resonance spectroscopy using an X-band EMS 104 ESR spectrometer (Bruker Co., Germany) with 2.50 mW microwave power, 6.37 G modulation amplitude, 100 G sweep width, 20 dB receiver gain, and 3440–3520 G scan range at room temperature.

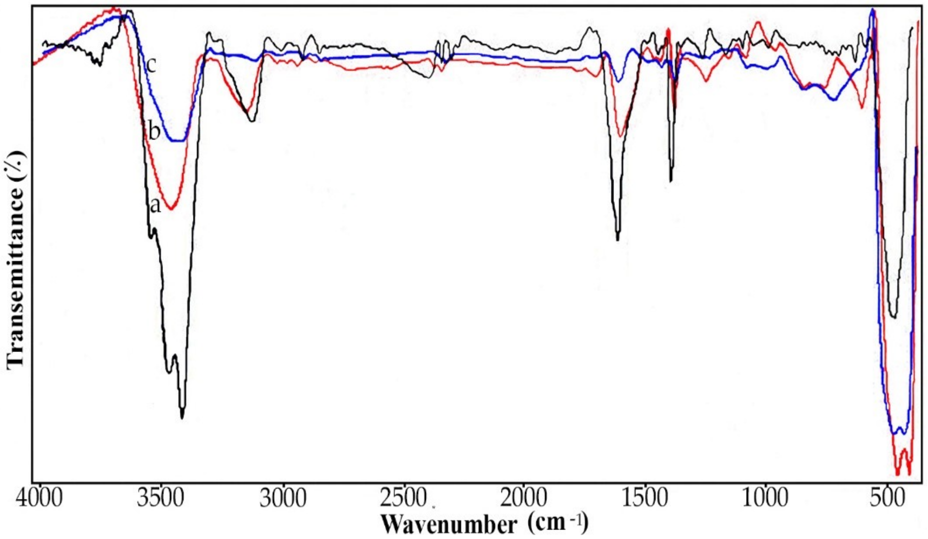

Fig. S1 FT-IR spectra of ZnO nanoparticles calcined at a) 400, b) 500 and c) 600 °C.

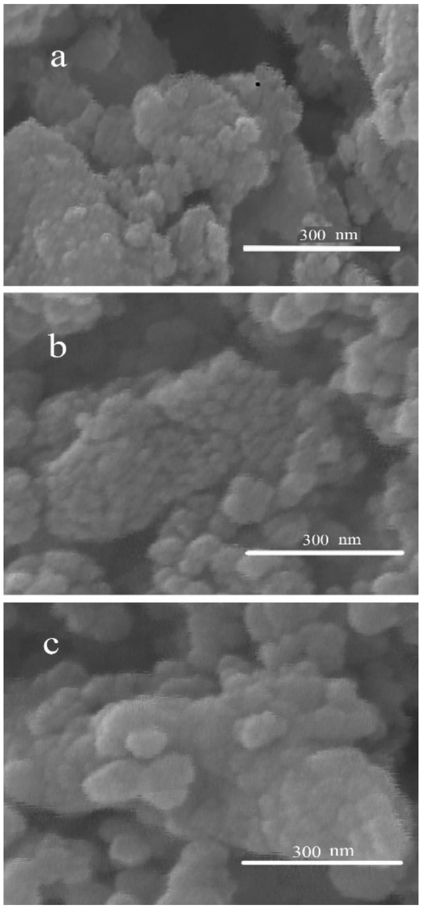

Fig. S2 SEM micrographs of ZnO nanoparticles calcined at a) 400, b) 500 and c) 600 °C.

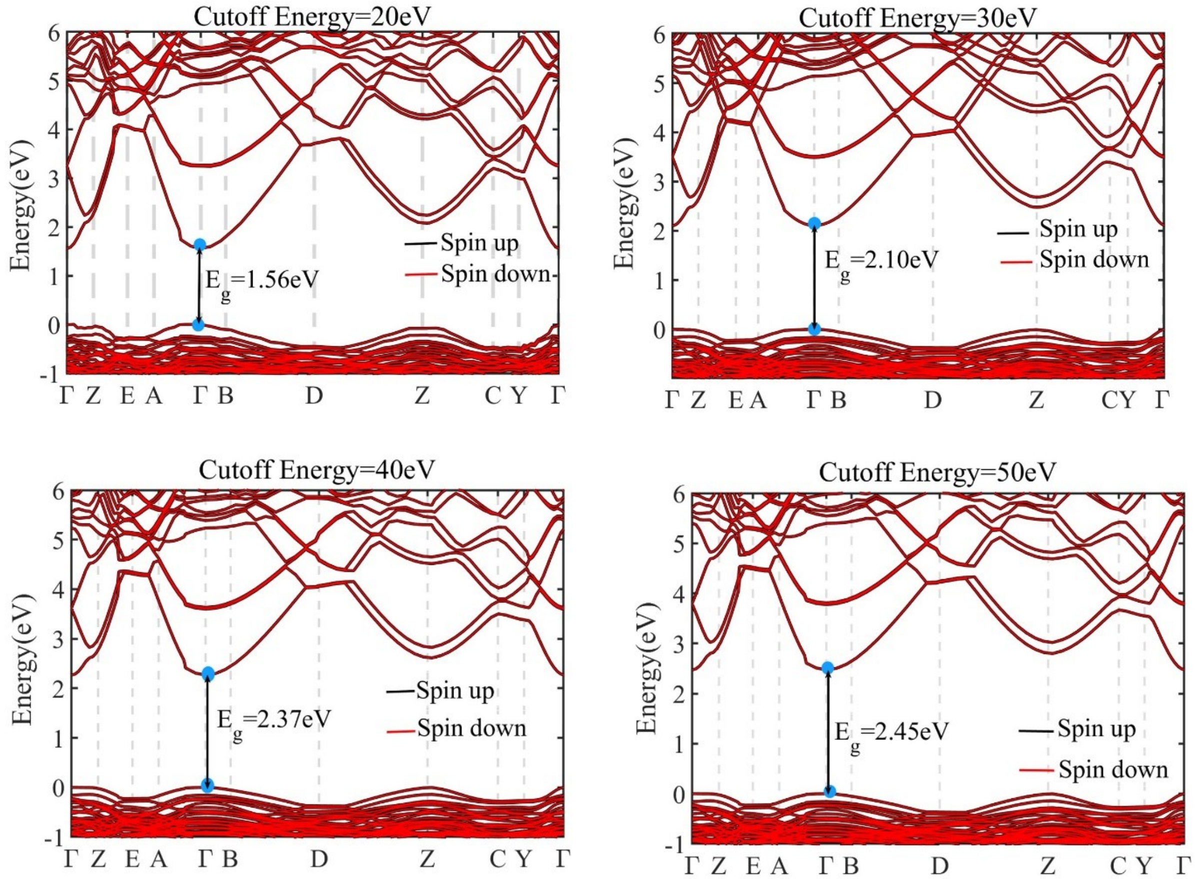

Fig S3. Convergence of energy cutoff for electronic band structures of pure ZnO (charge density of 160 Ry, a k-point grid of 2×3×3 (Monkhorst–Pack), Up(O) of 7 eV, and Ud(Zn) of 10 eV)

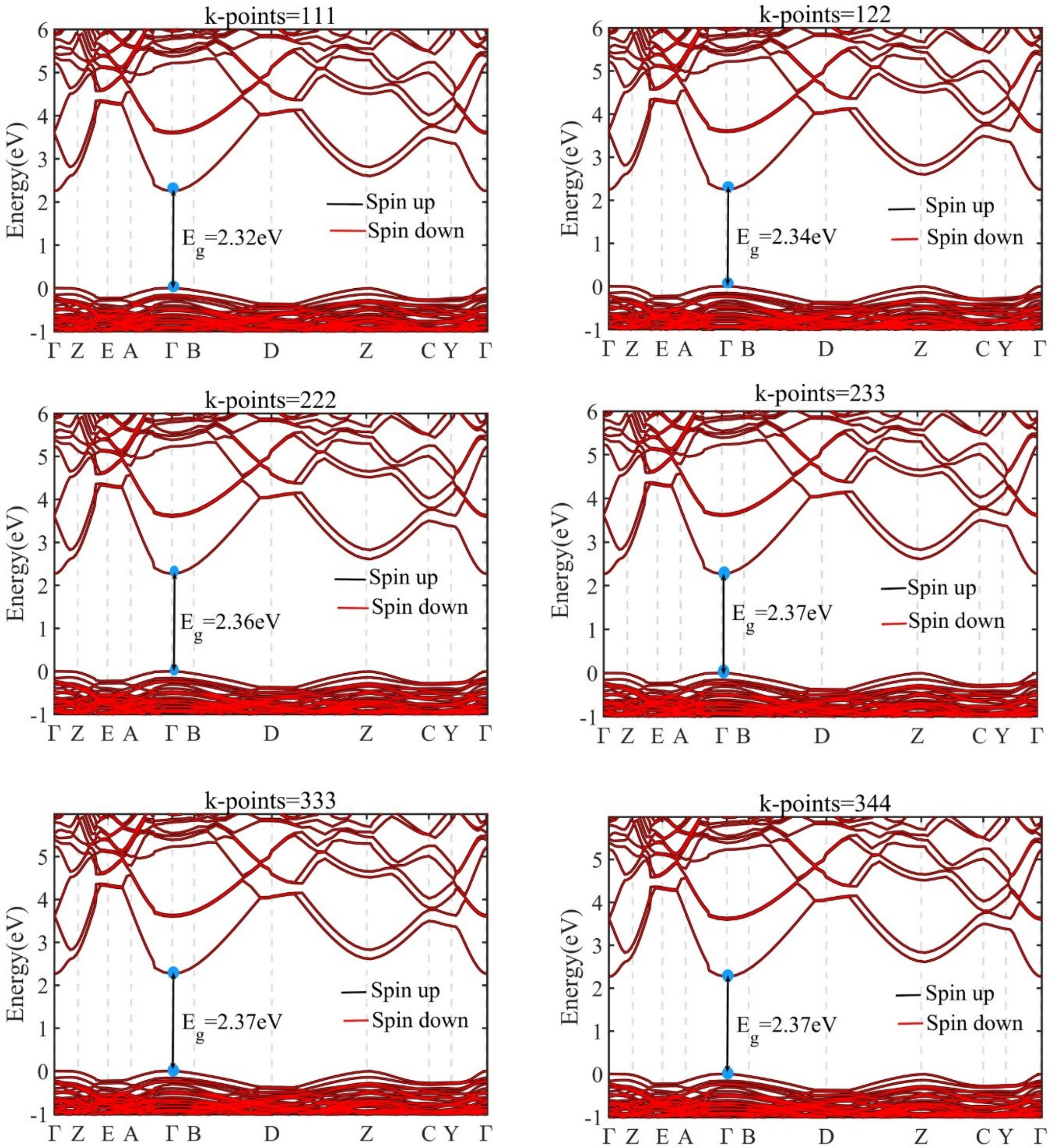

Fig S4. Convergence of k-point for electronic band structures of pure ZnO (energy cutoff of 40 Ry, a charge density of 160 Ry, Up(O) of 7 eV, and Ud(Zn) of 10 eV).

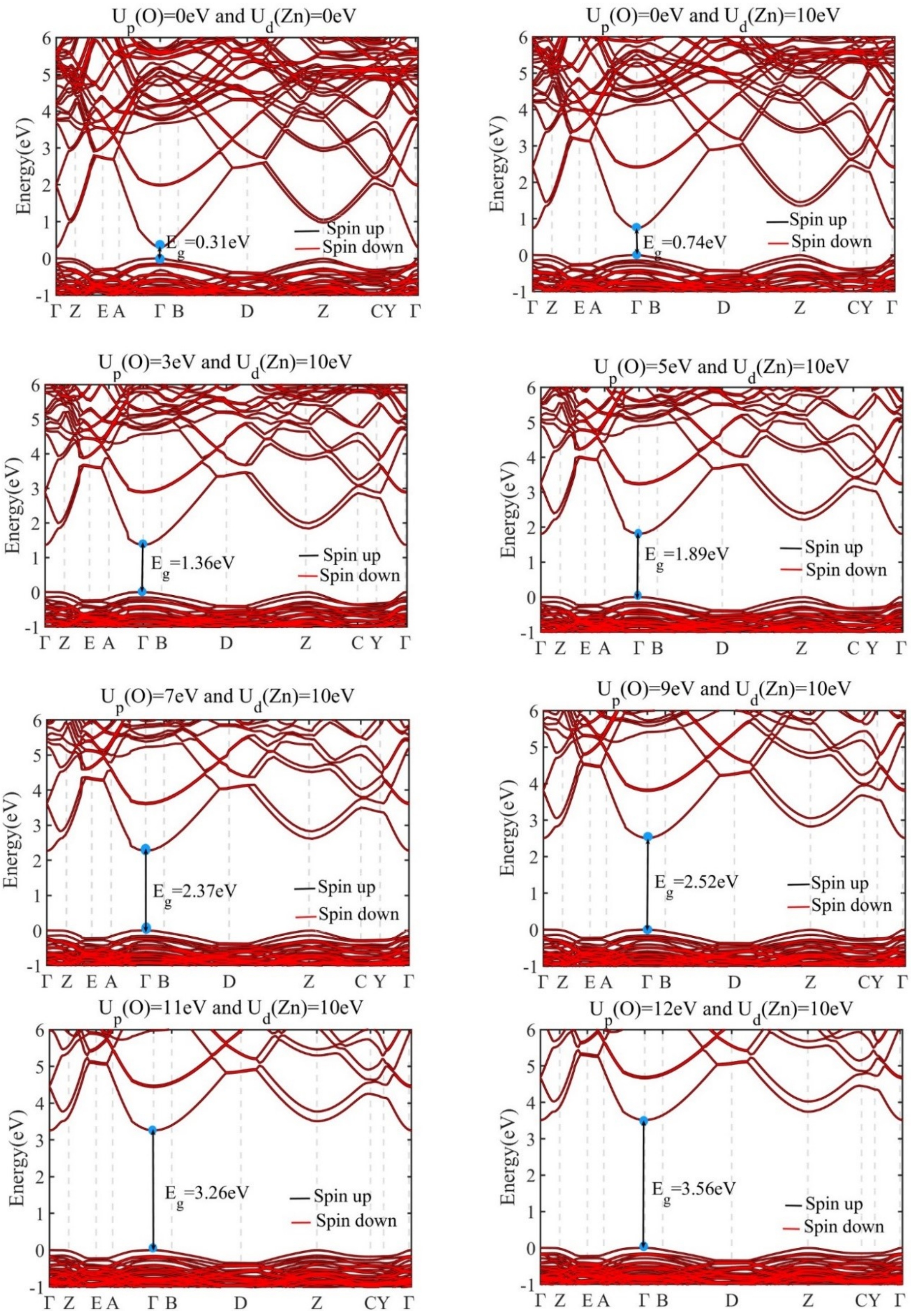

Fig S5. Effect of Hubbard Up(O) for electronic band structures of pure ZnO (energy cutoff of 40 Ry, a charge density of 160 Ry, a k-point grid of 2×3×3 (Monkhorst–Pack) and Ud(Zn) of 10 eV)

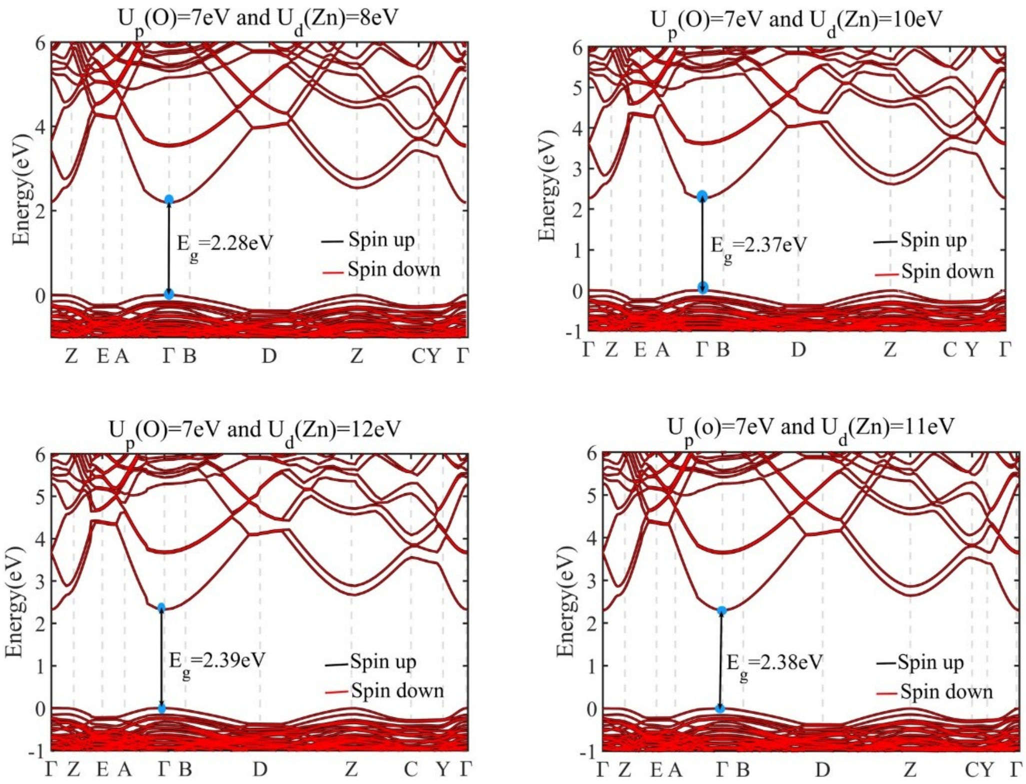

Fig S6. Effect of Hubbard Ud(Zn) for electronic band structures of pure ZnO (energy cutoff of 40 Ry, a charge density of 160 Ry, a k-point grid of 2×3×3 (Monkhorst–Pack), and Up(O) of 7 eV).

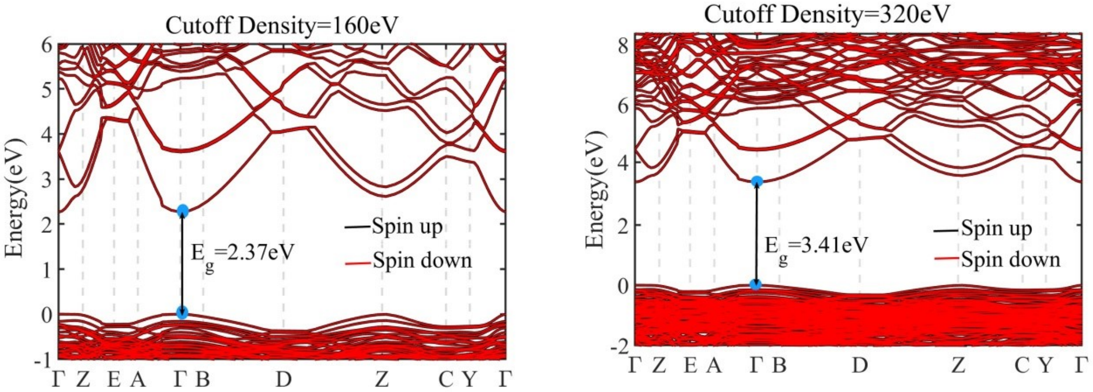

Fig S7. Effect of charge density for electronic band structures of pure ZnO (energy cutoff of 40 Ry, a k-point grid of 2×3×3 (Monkhorst–Pack), Up(O) of 7 eV, and Ud(Zn) of 10 eV).

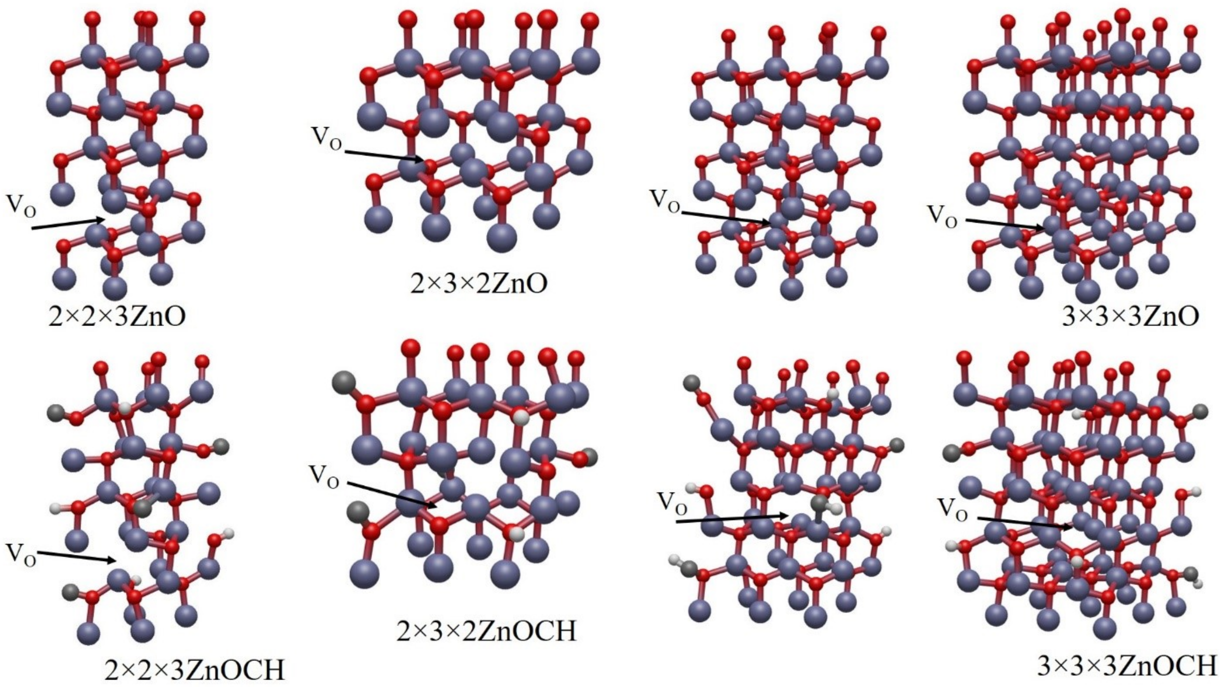

Fig S8. The 2×2×3, 2×3×2, 2×3×3 and 3×3×3 supercells of ZnO and ZnOCH with incorporating V_O defect.

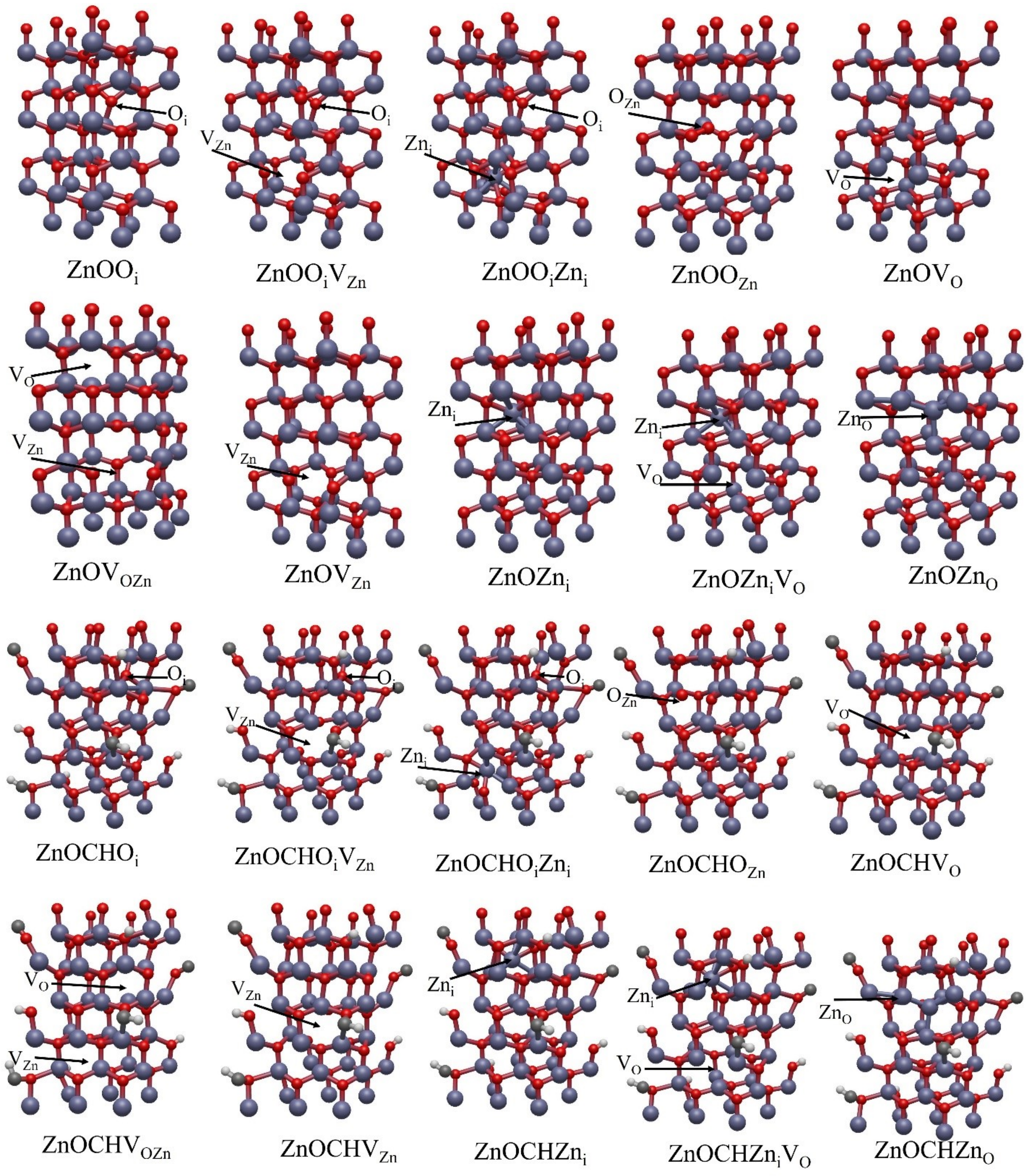

Fig S9. The supercells of ZnO and ZnOCH with incorporating native defects.

Table S1. Chemical potential of the atomic species Zinc and Oxygen under O-poor, O-rich and equilibrium conditions in eV.

	**O-poor**	**O-rich**	**Equilibrium**
**O**	-33.9277936341	-33.502152345	-33.714972
**Zn**	-148.146210335	-148.5718516241	-148.3590319

Table S2. The total energy and formation energy of the 2×2×3 supercell for pure ZnO structure and with incorporating V_O defect.

			**Formation Energy (eV)**		
	charge	Total Energy (Ry)	O-poor	O-rich	Equilibrium
**ZnO**	0	-4369.96625516	-	-	-
V_O	0	-4336.14600439	-1.46	4.32	1.43
	+1	-4336.66285093	-3.78	2.01	-0.88
	-1	-4335.55761908	1.83	7.62	4.72

Table S3. The total energy and formation energy of the 2×3×2 supercell for pure ZnO structure and with incorporating V_O defect.

			**Formation Energy (eV)**		
	charge	Energy (Ry)	O-rich	O-poor	Equilibrium
**ZnO**	0	-4369.81217179	-	-	-
V_O	0	-4336.01215931	4.05	-1.73	1.16
	+1	-4336.59727129	2.58	-3.20	-0.31
	-1	-4335.35841532	6.45	0.24	3.34

Table S4. The total energy and formation energy of the 3×3×3 supercell for pure ZnO structure and with incorporating V_O defect.

			**Formation Energy (eV)**		
	charge	Energy (Ry)	O-rich	O-poor	Equilibrium
**ZnO**	0	-9832.49850915	-	-	-
**VO**	0	-9798.62801538	-0.78	5.01	2.11
	+1	-9799.14730714	-2.12	3.67	0.77
	-1	-9798.05521194	1.29	7.08	4.18

Table S5. The total energy and formation energy of the 2×3×3 supercell for pure ZnO structure and with incorporating native defects.

	charge	Total Energy (Ry)	Formation Energy (eV)		
			O-poor	O-rich	Equilibrium
**ZnO**	0	-6554.66414289	-	-	-
O_i	0	-6587.97668226	8.37	2.58	5.47
	+1	-6588.43536696	8.10	2.31	5.21
	-1	-6587.43649919	9.75	3.95	6.85
O_iV_{Zn}	0	-6439.10919908	18.18	6.60	12.39
	+1	-6439.54072375	17.78	6.20	11.99
	-1	-6438.65879874	18.84	7.26	13.05
O_iZn_i	0	-6736.04888343	9.37	9.37	9.37
	+1	-6736.63093633	7.43	7.43	7.43
	-1	-6735.43451813	11.76	11.76	11.76
O_{Zn}	0	-6439.38439184	14.44	2.85	8.64
	+1	-6439.83636289	14.26	2.68	8.46
	-1	-6438.88395433	15.27	3.69	9.48
V_O	0	-6520.80057549	-0.87	4.91	2.02
	+1	-6521.30159714	-1.72	4.08	1.18
	-1	-6520.24938503	0.65	6.44	3.55
V_{OZn}	0	-6372.16889090	5.73	5.73	5.73
	+1	-6372.61619185	5.62	5.62	5.62

	-1	-6371.70456520	6.07	6.07	6.07
V_{Zn}	0	-6405.96110186	7.57	1.78	4.68
	+1	-6406.45684425	6.80	1.01	3.90
	-1	-6405.38449988	9.45	3.66	6.55
Zn_i	0	-6702.76868507	0.57	6.35	3.46
	+1	-6703.35134903	-1.39	4.41	1.61
	-1	-6702.15727975	2.91	8.70	5.80
Zn_iV_O	0	-6668.88397106	-0.02	11.56	5.77
	+1	-6669.47675745	-2.11	9.46	3.68
	-1	-6668.27175616	2.34	13.92	8.13
Zn_O	0	-6669.01002313	-1.73	9.84	4.05
	+1	-6669.57290311	-3.42	8.15	2.37
	-1	-6668.41939729	0.33	11.91	6.12

Table S6. The total energy and formation energy of the 2×2×3 supercell for pure ZnOCH structure and with incorporating V_O defect.

			Formation Energy (eV)		
	charge	Total Energy (Ry)	O-poor	O-rich	Equilibrium
**ZnOCH**	0	-4420.56346182			
V_O	0	-4386.74868928	-1.53	4.25	1.36
	+1	-4387.31489267	-4.53	1.26	-1.63
	-1	-4386.17410893	1.56	7.36	4.46

Table S7. The total energy and formation energy of the 2×3×2 supercell for pure ZnOCH structure and with incorporating V_O defect.

			Formation Energy (eV)		
			O-poor	O-rich	Equilibrium
	charge	Energy (Ry)	-	-	-
**ZnOCH**	0	-4410.52823622			
V_O	0	-4376.70038805	-1.35	4.43	1.54
	+1	-4377.38234592	-5.92	-0.14	-3.03
	-1	-4376.00590240	3.37	9.17	6.27

Table S8. The total energy and formation energy of the 3×3×3 supercell for pure ZnOCH structure and with incorporating V_O defect.

			Formation Energy (eV)		
	charge	Energy (Ry)	O-poor	O-rich	Equilibrium
**ZnOCH**	0	-9885.25554482			
V_O	0	-9851.32863506	-0.012	5.77	2.88
	+1	-9851.91312111	-2.24	3.80	0.78
	-1	-9850.73520006	2.34	7.88	5.11

Table S9. The total energy and formation energy of the 2×3×3 supercell for pure ZnOCH structure and with incorporating native defects.

			Formation Energy (eV)		
	charge	Energy (Ry)	O-poor	O-rich	Equilibrium
**ZnOCH**	0	-6607.02216146	-	-	-
O_i	0	-6640.64920967	4.09	-1.69	1.2
	+1	-6641.24732933	1.93	-3.86	-0.96
	-1	-6640.03973602	6.41	0.62	3.51
O_iV_{Zn}	0	-6491.51240056	17.56	5.98	11.77
	+1	-6492.07275158	15.91	4.34	10.12
	-1	-6490.94104942	19.37	7.79	13.58
O_iZn_i	0	-6788.07565580	13.88	13.88	13.88
	+1	-6788.68092968	11.61	11.61	11.61
	-1	-6787.46038935	16.28	16.28	16.28
O_{Zn}	0	-6491.95042267	11.61	0.027	5.81
	+1	-6492.54329033	9.51	-2.06	3.73
	-1	-6491.34620534	13.86	2.27	8.06
V_O	0	-6573.11695611	-0.30	5.48	2.59
	+1	-6573.72040639	-2.54	3.25	0.36
	-1	-6572.50399661	2.06	7.85	4.95
V_{OZn}	0	-6424.79932592	2.02	2.02	2.02
	+1	-6425.38447365	0.04	0.04	0.04

	-1	-6424.20086327	4.19	4.19	4.19
V_{Zn}	0	-6458.56930017	4.17	-1.61	1.28
	+1	-6459.14534876	2.31	-3.48	-0.59
	-1	-6457.98073197	6.21	0.41	3.31
Zn_i	0	-6755.09782291	0.95	6.75	3.85
	+1	-6755.72161179	-1.55	4.26	1.36
	-1	-6754.46403403	3.61	9.45	6.53
Zn_iV_O	0	-6720.94651315	4.00	15.58	9.79
	+1	-6721.56358663	1.58	13.15	7.36
	-1	-6720.31944189	6.56	18.14	12.35
Zn_O	0	-6721.42527972	-2.51	9.06	3.27
	+1	-6721.97703398	-4.05	7.53	1.74
	-1	-6720.80456681	-0.04	11.54	5.75
